# Deriving Membrane–Water and Protein–Water Partition Coefficients from In Vitro Experiments for Per- and Polyfluoroalkyl Substances (PFAS)

**DOI:** 10.1021/acs.est.4c06734

**Published:** 2025-01-06

**Authors:** Ruiwen Chen, Derek Muensterman, Jennifer Field, Carla Ng

**Affiliations:** † Department of Civil & Environmental Engineering, University of Pittsburgh, Pittsburgh, Pennsylvania 15261, United States; ‡ Department of Chemistry, Oregon State University, Corvallis, Oregon 97331, United States; § Department of Environmental and Molecular Toxicology, Oregon State University, Corvallis, Oregon 97331, United States

**Keywords:** PFAS, phospholipid membrane, human serum albumin, partition coefficients, in vitro, in silico

## Abstract

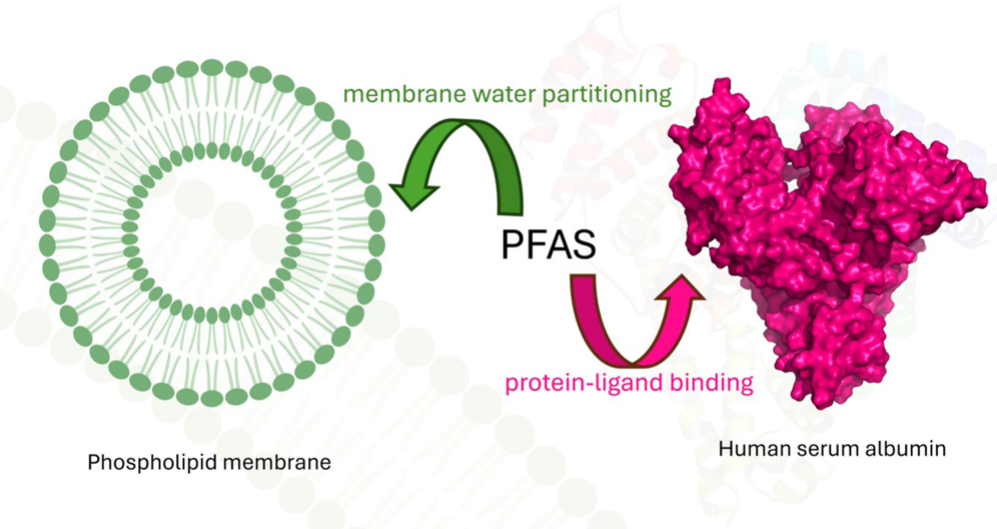

The phospholipid membrane–water partition coefficients (*K*_MW_) and equilibrium binding affinities for human serum albumin (HSA) of 60 structurally diverse perfluoroalkyl and polyfluoroalkyl substances (PFAS) were evaluated through laboratory measurements and modeling to enhance our understanding of PFAS distribution in organisms. Per- and polyfluoroalkyl carboxylic acids exhibited a 0.36 ± 0.01 log-unit increase in *K*_MW_ as the fluorinated carbon chain length increased from C_4_ to C_16_, while per- and polyfluoroalkyl sulfonates showed a 0.37 ± 0.02 log-unit increase. The highest HSA affinity range was observed between C_6_ and C_10_, with the following structural subclass order: per- and polyfluoroalkyl sulfonates ≈ ether sulfonic acids > polyfluoroalkyl carboxylic acids > fluorotelomer unsaturated carboxylic acids > phosphate diesters ≈ per- and polyfluoroether carboxylic acids. A comparison between association rate constants (K_A_) and HSA–PFAS molecular docking predictions with AutoDock Vina indicated that modeling could effectively predict the affinity of PFAS to HSA, especially for PFAS carbon chain lengths from C_4_ to C_10_. Based on in vitro results, exposure-dependent PFAS partitioning in organisms was modeled by comparing distribution coefficients between PFAS in phospholipid membranes and HSA at different PFAS concentrations and demonstrated that at lower concentrations, PFAS had higher partitioning in HSA, while with increasing concentration, the proportion of binding relative to the aqueous phase shifted toward the phospholipid membrane. Few studies have compared the bioaccumulation of PFAS in phospholipid membranes and HSA. This research reports that protein–water distribution coefficients are higher than membrane–water partitioning coefficients at lower PFAS concentrations, which may have implications for interpreting exposure data and toxicity experiments.

## Abstract 2

The phospholipid membrane–water partition coefficients (*K*_MW_) and equilibrium binding affinities for human serum albumin of 60 structurally diverse perfluoroalkyl and polyfluoroalkyl substances were evaluated through laboratory measurements and modeling to enhance our understanding of PFAS distribution in organisms.

## Introduction

Per- and polyfluoroalkyl substances (PFAS) are ubiquitous environmental contaminants that have become pervasive in daily life, infiltrating the human body through various pathways^1−3^ to become widely distributed in tissues.^4^ Due to the similarity between the structure of PFAS and endogenous molecules, PFAS are known to interact with diverse transporters, intra- and extracellular proteins, nucleic acid receptors, and cell membranes.^5^ Recent studies modeled the accumulation or distribution of PFAS in the body through the use of pseudo bioconcentration factors^6^ or tissue-sorption capacity.^7^ More in-depth models were developed to facilitate better estimation of PFAS distribution by integrating cell membrane permeation, binding with specific proteins such as human serum albumin (HSA), and active transport facilitated by organic anion transporters.^8^ However, modeling partition or distribution for structurally diverse PFAS—such as polyfluorinated, unsaturated, ether-containing, chlorinated, cyclic, and phosphoric acid–based—needs further validation through laboratory measurements.

Phospholipids and serum albumin are thought to be two of the primary mediators of PFAS bioaccumulation and tissue distribution. Phospholipids, crucial constituents of cellular membranes, are believed to exert a significant influence on the bioaccumulation for anionic PFAS through their role in facilitating the transfer of anions from the aqueous phase to the lipid phase.^9^ HSA serves as the primary carrier protein in the blood and is also found in the interstitial fluid of human tissues.^10^ Research on the binding of PFAS to HSA dates back to the 1950s.^11^ However, it is only recently that the persistence and toxicity of PFAS triggered comprehensive studies on these compounds for their interactions with serum albumin and phospholipids.^9,12−14^

Artificial phospholipid membranes have been used to study the role of PFAS in tissue distribution, cell membrane penetration, and implications for toxicity.^15,16^ Solid-supported lipid membranes (SSLM) were used to measure phospholipid membrane–water partition coefficients (*K*_MW_) for perfluoroalkyl carboxylic acids (PFCAs) and perfluoroalkyl sulfonate acids (PFSAs).^17^ However, it is important to note that not all PFAS compounds exhibit the same interactions with phospholipids; interactions may be influenced by whether they are short-chain, long-chain, zwitterionic, cationic, or anionic.

The binding affinity between HSA and PFAS is quantified using either an equilibrium association constant (*K*_A_) or a dissociation constant (*K*_D_, the inverse of *K*_A_). Equilibrium dialysis, an in vitro method considered a gold standard for evaluating *K*_D_, is performed using a variety of commercially available systems including two-chamber 96-well systems,^6^ Rapid Equilibrium Dialysis^18^ (RED), and Slide-A-Lyzer^19^ systems, which have achieved consistent trends for PFAS–protein interactions.^5,6,18,20^ However, while binding trends determined using various methods are consistent,^21^ the values of HSA binding affinities vary greatly,^5^ making it challenging to compare binding strengths across structures when measured using different methods. Hence, in vivo experiments still provide critical information on PFAS distribution within tissues in the absence of comprehensive and reliable in vitro data sets that can enable more robust extrapolation or prediction to different systems or PFAS.

Protein–ligand docking, often employed as a rapid screening tool, is used to identify interactions between PFAS and proteins.^22^ In addition to affinity estimation, the bound protein–ligand conformations derived from docking simulations are adopted for qualitative comparisons,^6^ to identify the structural components of PFAS that exhibit the highest affinity for the binding site of the protein. Binding conformations obtained from docking are used to infer the varying affinities of the binding sites for ligands of interest and potentially identify reasons for discrepancies between docking predictions and experimental results.^5,18,22^

In this work, we investigated the distribution of PFAS to phospholipid membranes and HSA from the aqueous phase via laboratory measurements with 60 PFAS and subsequent modeling. Phospholipid membrane–water partition coefficients (*K*_MW_) were measured with SSLM and specific binding to HSA was estimated by equilibrium dialysis. Then, the specific-binding curves from equilibrium dialysis were extrapolated to simulate the PFAS HSA/Water distribution (*D*_HSA/W_). The binding sites of PFAS on HSA were analyzed by using molecular docking. Subsequently, we compared *K*_MW_ and *D*_HSA/W_ at different aqueous concentrations of PFAS to evaluate how the relative distribution of PFAS to phospholipid membranes versus HSA changed with concentration.

## Materials and Methods

### Chemicals and Materials

All PFAS standards (Table 1) were purchased from Wellington (Guelph, Ontario, Canada). Each compound’s acronym and Chemical Abstracts Service Registry Number (CASRN) are listed in the Supporting Information Table S1. The 60 target PFAS for measurements were divided into groups according to their structures (Tables 1 and S2). Deionized water was produced from a Milli-Q (Burlington, MA, USA) water purification system. Methanol, ammonium acetate, ammonium hydroxide, and acetic acid were of LC/MS grade and purchased from ThermoFisher (Hanover Park, IL, USA). The phospholipid membrane–water partition coefficients were evaluated using a commercial solid-supported lipid membrane (SSLM) kit, the TRANSIL Membrane Affinity Kit (Sovicell GmBH, Leipzig, Germany). All centrifuge tubes and LC/MS sample vials used for measurements were made of polypropylene. Purified HSA (molecular weight 66.4 kDa, 10 mg/mL solution) was purchased from MilliporeSigma (Burlington, MA, USA). Slide-A-Lyzer mini dialysis devices with 10k molecular weight cutoff (10k MWCO, 0.1 mL) were purchased from Fisher Scientific (Hanover Park, IL, USA). Buffers were prepared from ammonium acetate with the pH adjusted by ammonium hydroxide or acetic acid depending on the pH requirements.

**Table 1:** Names and Abbreviations of PFAS, Group Names, and Acronyms Based on Structure^a^

group name	PFAS
perfluoroalkyl carboxylic acids (PFCA)	perfluorobutanoic acid (PFBA); perfluoropentanoic acid (PFPeA); perfluorohexanoic acid (PFHxA); perfluoroheptanoic acid (PFHpA); perfluorooctanoic acid; perfluorononanoic acid (PFOA); perfluorodecanoic acid (PFDA); perfluoroundecanoic acid^b^ (PFUnA); perfluorododecanoic acid^b^ (PFDoA); perfluorotridecanoic acid^b^ (PFTrDA); perfluorotetradecanoic acid^b^ (PFTeDA); perfluorohexadecanoic acid^b^ (PFHxDA)
perfluoroalkyl sulfonic acids (PFSA)	perfluorobutanesulfonic acid (PFBS); perfluoropentansulfonic acid (PFPeS); perfluorohexanesulfonic acid (PFHxS); perfluoroheptanesulfonic acid (PFHpS); perfluorooctanesulfonic acid (PFOS); perfluorononanesulfonic acid (PFNS); perfluorodecanesulfonic acid^b^ (PFDS); perfluorododecanesulfonic acid^b^ (PFDoS)
fluorotelomer sulfonic acids (FTS)	1*H*,1*H*,2*H*,2*H*-perfluorohexanesulfonic acid (4:2 FTS); 1*H*,1*H*,2*H*,2*H*-perfluorooctanesulfonic acid (6:2 FTS); 1*H*,1*H*,2*H*,2*H*-perfluorodecanesulfonic acid (8:2 FTS); 1*H*,1*H*,2*H*,2*H*-perfluorododecanesulfonic acid^b^(10:2 FTS)
perfluorooctane sulfonamides (PFOSAm)	perfluorooctanesulfonamide^b^ (PFOSA); *N*-methyl perfluorooctanesulfonamide (NMeFOSA); *N*-ethyl perfluorooctanesulfonamide (NEtFOSA)
perfluorooctane sulfonamidoacetic acids (PFOSAA)	*N*-methyl perfluorooctanesulfonamidoacetic acid (NMeFOSAA); *N*-ethyl perfluorooctanesulfonamidoacetic acid (NEtFOSAA); perfluorooctanesulfonamidoacetic acid (FOSAA)
,perfluorooctane sulfonamide ethanols (PFOSE)	*N*-methyl perfluorooctanesulfonamidoethanol (NMeFOSE); *N*-ethyl perfluorooctanesulfonamidoethanol (NEtFOSE)
per- and polyfluoroether carboxylic acids (PFECA)	hexafluoropropylene oxide dimer acid (HFPO-DA); 4,8-dioxa-3*H*-perfluorononanoic acid (ADONA); perfluoro-3-methoxypropanoic acid (PFMPA); perfluoro-4-methoxybutanoic acid (PFMBA); Nonafluoro-3,6-dioxaheptanoic acid (NFDHA)
ether sulfonic acids (ESA)	9-chlorohexadecafluoro-3-oxanonane-1-sulfonic acid^b^(9Cl-PF3ONS); 11-chloroeicosafluoro-3-oxaundecane-1-sulfonic acid^b^(11Cl-PF3OUdS); perfluoro(2-ethoxyethane)sulfonic acid (PFEESA)
fluorotelomer carboxylic acids (FTCA)	3-perfluoropropyl propanoic acid (3:3 FTCA); 2*H*,2*H*,3*H*,3*H*-perfluorooctanoic acid (5:3 FTCA); 3-perfluoroheptyl propanoic acid (7:3 FTCA); 2-(perfluorohexyl)ethanoic acid (6:2 FTCA); 2-(perfluorooctyl)ethanoic acid (8:2 FTCA); 2-(perfluorodecyl)ethanoic acid (10:2 FTCA)^b^
fluorotelomer unsaturated carboxylic acid (FTUCA)	6:2 fluorotelomer unsaturated carboxylic acid (6:2 FTUCA); 8:2 fluorotelomer unsaturated carboxylic acid (8:2 FTUCA)
phosphate diester (diPAP)	6:2 fluorotelomer phosphate diester^b^(6:2 diPAP); 8:2 fluorotelomer phosphate diester^b^(8:2 diPAP); EtFOSE-based phosphate diester (diSAmPAP)^b^
other PFAS	8-chloro-perfluorooctanesulfonic acid (8Cl-PFOS); perfluoro-*p*-ethylcyclohexylsulfonic acid (PFEtCHxS); perfluorobutanesulfonamide (FBSA); perfluorohexanesulfonamide (FHxSA); perfluorohexane sulfonamido amine (PFHxSaAm,); 6:2 fluorotelomer sulfonamide betaine (6:2 FtSaB); N-trimethylammoniopropyl perfluorohexane sulfonamide (N-TAmP-FHxSA); 5:3 fluorotelomer betaine (5:3 FTB); 5:1:2 fluorotelomer betaine (5:1:2 FTB)

a Those that cannot be categorized based on their structure or with too few chemicals are grouped under “Other PFAS”.

b Indicates these are highly hydrophobic members of their respective groups; their *K*_MW_ were measured with additional steps to account for nonspecific binding to assay components.

## *K*_MW_ on Solid Supported Phospholipid Bilayers

Membrane–water partition coefficients (*K*_MW_) were measured using SSLM following published experimental procedures^17^ that are described in the Supporting Information. In brief, the phosphatidylcholine membrane beads (lipid volumes from 0.067 to 2.166 μL) from the membrane affinity kit were transferred into 1.5 mL centrifuge vials, followed by exchanging the original buffer with 10 mM ammonium acetate, which eliminated salt crystals. Subsequently, test vials in each series were spiked with the same level for each PFAS group and then equilibrated on a shaker at 50 rpm for 4 h. The supernatants were then transferred to polypropylene vials for analysis after centrifuging at 10,000*g* for 10 min. For the highly hydrophobic PFAS (denoted by subscript “*b*” in Table 1), an additional container surface rinse with methanol was analyzed and the measured amount added to what was detected in the supernatant. The PFAS were analyzed to determine *K*_MW_ at pH6 and pH7, in triplicate.

### Equilibrium Dialysis on Slide-A-Lyzer Dialysis Devices

The equilibrium dialysis experiments followed methods previously described.^19^ In brief, PFAS to HSA molar ratios of 1:16, 1:8, 1:4, 1:2, 1:1, and 2:1 were used in each dialysis experiment. PFAS solutions (1.0 mL) were added to 1.5 mL microcentrifuge sampling vials, which were then fitted with 10,000 MWCO dialysis cups. Then, 100 μL of 1.0 μM HSA was added to the dialysis cup, and the samples were capped and equilibrated on a shaker for 48 h. Controls included PFAS standards without HSA and blank samples with HSA but without PFAS. All experiments were run in duplicate. After 48 h, the caps were removed and liquid below the dialysis cup was transferred for analysis by liquid chromatography tandem mass spectrometry.

### Analytical Method

The supernatant of the SSLM vials and the dialysate (free PFAS in solution after equilibration in the dialysis system) were analyzed on a ThermoFisher Vanquish UHPLC coupled to a Quantis tandem mass spectrometer (Waltham, MA, USA). External calibration standards were used to quantify the initial concentration of all of the spikes. After the SSLM partitioning experiment, the samples were quantified by employing the mean area of the quantified ions in the spiked sample. For equilibrium dialysis assessments, the samples were quantified utilizing the same spike sample to compensate for any nonspecific binding. Both experimental and laboratory blanks were also assessed throughout the study, with all values falling below the minimum level of quantification. Chromatographic separation parameters and mass transitions are provided in Supporting Information Table S3.

### Molecular Docking for HSA

To understand how binding conformations contribute to the observed affinity of different PFAS for HSA, we performed molecular docking using AutoDock Vina v.1.1.2 on a Linux x86_64 operation system.^23,24^ A nine-grid box, each box measuring 26 × 26 × 26 Å with a spacing of 1.0 Å, was employed to encompass the entire HSA structure for the docking procedure (Table S4). Thus, the main fatty acid (FA) and drug-binding sites of HSA were covered, and other binding sites could also be detected.^25^ The crystal structure of HSA (Protein Data Bank ID: 1AO6(26)) was selected for good resolution (2.5 Å) and positively charged residues located at similar positions in the subdomain of known binding sites. The Simplified Molecular Input Line Entry System (SMILES) of PFAS molecules was first extracted from the CompTox Chemistry dashboard;^27^ then the acid groups were deprotonated to yield their anion or zwitterionic forms and saved as mol2 files. The three-dimensional structures of PFAS ligands were then prepared from mol2 to PDBQT for simulation with the python Meeko package.^28^ The HSA structure was prepared first by adding the correct protonation state for the specific pH with the Adaptive Poisson–Boltzmann Solver (APBS) software.^29^ The Lamarckian genetic algorithm^30^ was then applied to seek the best binding site for PFAS in HSA with parameters. Exhaustiveness of the global search was set to 32 and maximum number of binding modes to generate was set to 20 to study the conformations of docking results, of which the conformation with the lowest binding energy was selected and analyzed using Pymol (New York, NY, USA). The equilibrium association constant, *K*_A_, was calculated from the Gibbs free energy (Δ*G*) relationship: Δ*G* = −*RT*ln *K*_A_.

### PFAS Distribution in HSA/Water Estimation

In this work, the in vitro HSA binding was extrapolated to the in vitro distribution of compounds between HSA and water based on the relationship between HSA binding and drug distribution.^31,32^ We assume that the distribution of PFAS between HSA and water changes with the concentration in the aqueous phase, according to the HSA binding affinity curve. First, the HSA and PFAS ligand interact forming a 1:1 complex at equilibrium
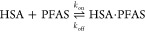
1

Then, the equilibrium association constant (*k*_on_, on-rate constant) and equilibrium dissociation constant (*k*_off_, off-rate constant) are defined as
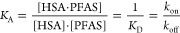
2

For HSA, PFAS total specific binding with multiple sites follows the Langmuir adsorption isotherm^33^
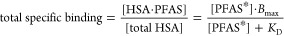
3where [PFAS*] is the PFAS concentration in the aqueous phase at equilibrium and *B*_max_ is the total number of binding sites derived from the specific binding curve of a single site. To obtain the distribution coefficients, we extrapolated the binding curves, assuming single-site binding using equilibrium dialysis.
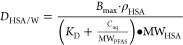
4where *D*_HSA/W_ is the distribution coefficient (pseudounitless) of PFAS between HSA and water under specific conditions; ρ_HSA_ is the density of HSA in g/L; *K*_D_ is the equilibrium dissociation constant in mol/L (PFAS mol/water L); *C*_aq_ is the concentration of PFAS in the water phase in g/L; MW_PFAS_ is the molecular weight of PFAS in g/mol; and *MW*_HSA_ is the molecular weight of HSA in g/mol.

## Results and Discussion

### General Observations on Phospholipid Membrane Partitioning

Phospholipid membrane–water partition coefficients were measured for structurally diverse PFAS, including fluorotelomer unsaturated carboxylic acids, chlorinated polyfluorinated PFAS, cyclic carbon chain sulfonic acids, and fluorotelomer phosphate diesters, for which we provide some of the first measured values. We observed that varying structures lead to significant differences in *K*_MW_ compared with the recently studied PFCAs and PFSAs,^17^ even when the type of acidic head groups and number of fluorinated carbon atoms (FC_*n*_) remain the same.

**Figure 1:**
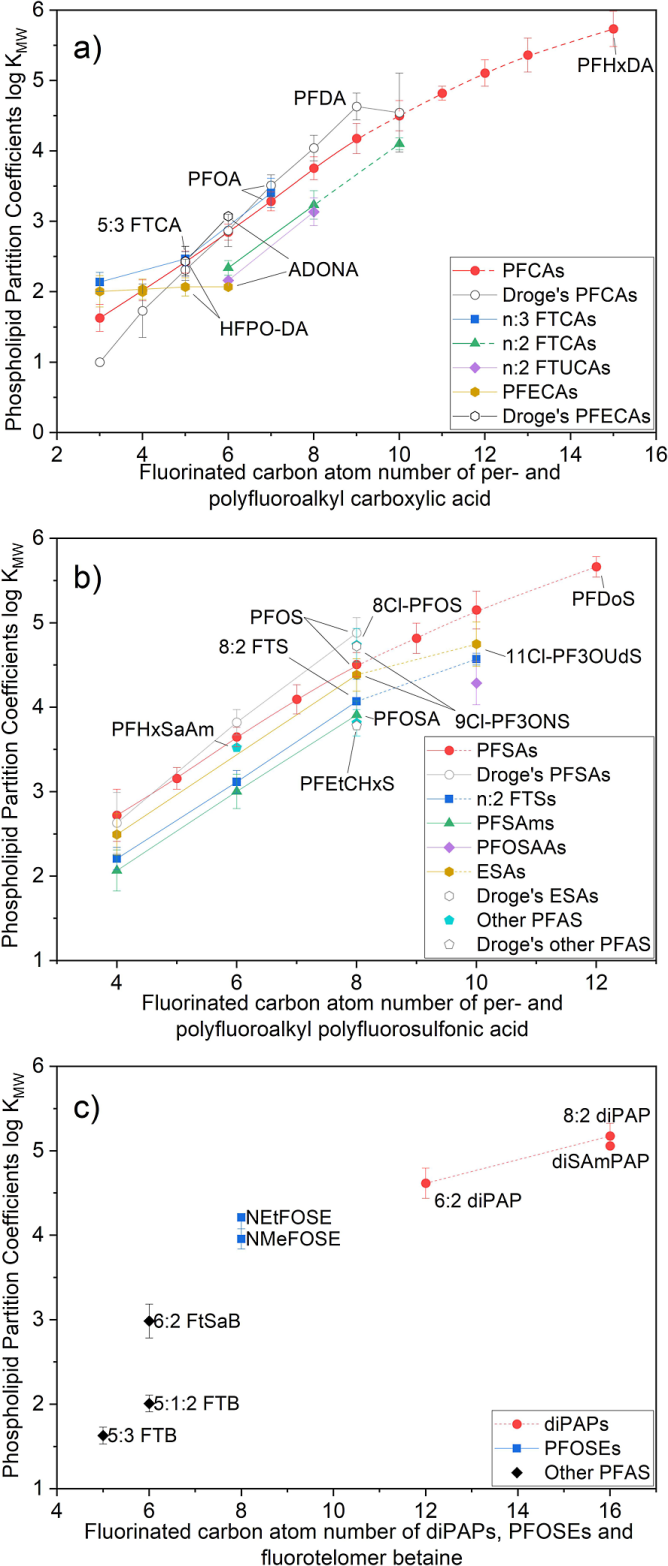
Log *K*_MW_ as a function of fluorinated carbons numbers (FC_*n*_), grouped based on the PFAS headgroup (carboxylic or sulfonic—there are more than these two major classes in this figure). The dashed line indicates that these PFAS have low solubility in water, and *K*_MW_ was calculated with eq S2 taking the measured residual on the container internal surfaces. (a) PFCAs, FTCAs, FTUCAs, and PFECAs. (b) PFSAs, *n*:2 FTSs, PFOSAms, PFSOAAs, and ESAs. (c) diPAPs, PFOSEs not defined. Compounds that do not fit these classes are individually labeled on the plots as Other PFAS.

The *K*_MW_ increases from FC_3_ to FC_16_, which indicates that increasing the chain length leads to higher partitioning to the membranes. For PFAS with the same number of fluorinated carbon atoms, polyfluoroalkyl substances with a larger number of total carbons do not have higher partition coefficients than their perfluoroalkyl counterparts, such as 8:2 fluorotelomer carboxylic acids (FTCA) < perfluorononanoic acid (PFNA) (Figure 1a) and 8:2 fluorotelomer sulfonic acids (FTS) < perfluorooctanesulfonic acid (PFOS) (Figure 1b) for the *K*_MW_ measurements.

### Per- and Polyfluoroalkyl Carboxylic Acid *K*_MW_ Based on Carbon Chain Length

The *K*_MW_ trend, as the fluorinated carbon numbers (FC_*n*_) increase from FC_3_ (perfluorobutanoic acid) to FC_15_ (perfluorohexadecanoic acid), remains consistent with recently published values for FC_3_ to FC_10_ (Figure 1a).^17^ The linear regression analysis indicates a 0.36 ± 0.01 log-units (base 10) increase in *K*_MW_ for each unit increase of CF_2_ for PFCAs, suggesting that longer fluorinated carbon chains promote the compound’s embedding into phospholipid membranes. The assessment method for the membrane–water partition coefficient requires measuring the concentration in the aqueous phase and calculating the concentration in the phospholipid membrane by difference (eq S1). That is, the amount of PFAS partitioned into the phospholipid membrane is determined by the difference in concentration in the water based on the amount originally added to the system and at the end of the experiment. However, adsorption of PFAS to the container surface could lead to a decrease in the final measured aqueous concentration in the phospholipid–water system for PFAS with carbon chains longer than FC_10_. Thus, an additional step was used to offset the PFAS adsorbed onto the container surface; at the end of the experiment, the assay vial was rinsed with methanol and analyzed for PFAS, and this amount was combined with the PFAS mass measured in the water phase to calculate the “unbound” PFAS. The difference between the initial added PFAS and this total (aqueous plus surface-sorbed) PFAS is the mass in the phospholipid phase.

The *K*_MW_ for per- and polyfluorinated PFAS follows the order: *n*:3 FTCAs > PFCAs > *n*:2 FTCAs > *n*:2 FTUCAs for the same number of fluorinated carbons. The sequence indicates that the *K*_MW_ of 7:3 FTCA is slightly higher than that of perfluorooctanoic acid (PFOA). However, for polyfluorinated carboxylic acids with the same FC_*n*_ as PFNA, 8:2 FTCA (one more CH_2_) and 8:2 FTUCA (one unsaturated CF=CH in addition to PFNA), the longer chain length does not increase *K*_MW_. Longer-chain PFAS may require more time to reach equilibrium between the phospholipid membrane and the water phase. The SSLM assay comprises six different phospholipid test vials ranging from low to high concentrations in which phospholipids are affixed to bead supports; thus, higher concentration vials have, correspondingly, increasing amounts of beads. The larger volume of the beads will lead to more collisions during the buffer change and shaking process. Consequently, beads may be directly exposed to water when the membrane is lost during these collisions. The bare solid–water interfaces on the beads may facilitate nonspecific binding of long-chain PFAS, potentially causing a reduction in the slope of the data used for calculating *K*_MW_. For PFECAs (PFAS containing ether bonds), the *K*_MW_ results do not show an increase with the number of fluorinated carbon atoms from FC_3_ to FC_6_, suggesting that the ether bond in the carbon chain may reduce membrane binding affinities compared to PFCAs.

### Per- and Polyfluoroalkyl Sulfonic Acid *K*_MW_ Based on Carbon Chain Length

For PFSAs, the *K*_MW_ increased 0.37 ± 0.02 units for each CF_2_ (Figure 1b), while Droge^17^ reported an increase of 0.53 units for FC_4_ to FC_8_ PFSA. For the per- and polyfluoroalkyl sulfonic acids with the same number of fluorinated carbon atoms, the log *K*_MW_ of fluorotelomer sulfonates (*n*:2 FTS) is lower than that of PFSA. The two extra CH_2_ separating the sulfonic acid head from the fluorinated carbon tail in *n*:2 FTS will contribute an increase of p*K*_a_ (logarithmic form of acid dissociation constant) by 4 units when compared to PFSA,^34^ which lowers the acidity of the headgroup. A longer carbon chain with a decreased hydrophilic acid group will not lead to a stronger *K*_MW_, consistent with the carboxylic acid group outcomes. Thus, the greater acidity from the perfluoroalkyl acids, leading to more hydrophilic heads, significantly contributes to these compounds’ stronger phospholipid membrane affinities relative to polyfluoroalkyl acids.

Sulfonamides, which can diffuse into the phospholipid membrane in their neutral form,^35^ are weaker acids compared to sulfonic acids^36^ and were observed to have a lower membrane affinity than *n*:2 FTS. According to the solubility-diffusion mechanism, PFAS partitioning to the phospholipid membrane encounters two barriers: the alkyl portion within the membrane and the interface where the zwitterions of the phospholipid’s polar headgroup meet water.^37^ Therefore, when compared to strongly acidic fluorotelomer sulfonates, weakly acidic sulfonamides with an identical fluorinated carbon tail would have similar abilities to dissolve into the hydrophobic part of the phospholipid membrane.

### DiPAP, Sulfonamide, Fluorotelomer Betaine PFAS *K*_MW_

The phosphoric acid headgroup PFAS studied were 8:2 diPAP, 6:2 diPAP, and the EtFOSE-based diPAP (Figure 1c). With an additional (CF_2_)_2_ on each side, the diPAP class of PFAS possesses two polyfluorinated carbon chains. We found that 8:2 diPAP demonstrated a greater *K*_MW_ than 6:2 diPAP; the log *K*_MW_ increased from 4.1 to 4.4. Comparing the FC_8_ FTCA and FTS to 8:2 diPAP, we found 8:2 diPAP > 8:2 FTS > 8:2 FTCA. Not surprisingly, diphosphates containing double the number of fluorinated carbon atoms exhibit stronger phospholipid membrane affinity than the PFAS-sulfonic acid and carboxylic acid groups. However, diSAmPAP with sulfonamide branches might exhibit a *K*_MW_ slightly lower than that of 8:2 diPAP due to the EtFOSEs linkage to the sulfonamide, which potentially reduces the overall polarity of the phosphate group. The fluorotelomer betaines, 5:3 FTB and 5:1:2 FTB, demonstrated slightly lower *K*_MW_ than the same carbon chain 5:3 FTCA, and 6:2 FtSaB was lower than 6:2 FTS. The hydrophilic group −N^+^(CH_3_)_2_–CH_2_–COO^–^, which can act as both a hydrogen donor and an acceptor, renders the molecule electrically net-neutral. The fraction between the zwitterionic and noncharged neutral of the compound might be a reason for its reduced partition coefficient.^38^ However, the molecular partitioning behavior for FTBs and FtSaBs to the phospholipid membrane requires further study.

### Measurement Results for *K*_MW_ at pH6 and pH7

Given that the phospholipid bilayers that are implicated in PFAS tissue permeation and accumulation are distributed throughout the body, the pH of fluids in specific tissues may also influence the partitioning of PFAS between the phospholipid and aqueous phases. Consequently, we compared outcomes at two pH levels: pH6 and pH7. Although the results show minor differences, those differences are not consistent across PFAS with measurable *K*_MW_ (Figure S1). For instance, the *K*_MW_ of PFOA (strong acid) at pH7 is elevated compared to that at pH6, whereas the *K*_MW_ values for *N*-methyl perfluorooctanesulfonamidoethanol and *N*-ethyl perfluorooctanesulfonamidoethanol (weak acids) at pH6 are marginally higher than those at pH7. Given that all other experimental conditions are the same, this discrepancy suggests that pH could potentially alter the ionic strength at the surface of the phospholipid membrane,^39,40^ the membrane viscosity,^41^ and the electron surface charge densities for PFAS,^42^ jointly influencing PFAS distribution between the two phases.

### Equilibrium HSA–PFAS Binding Affinity

**Figure 2:**
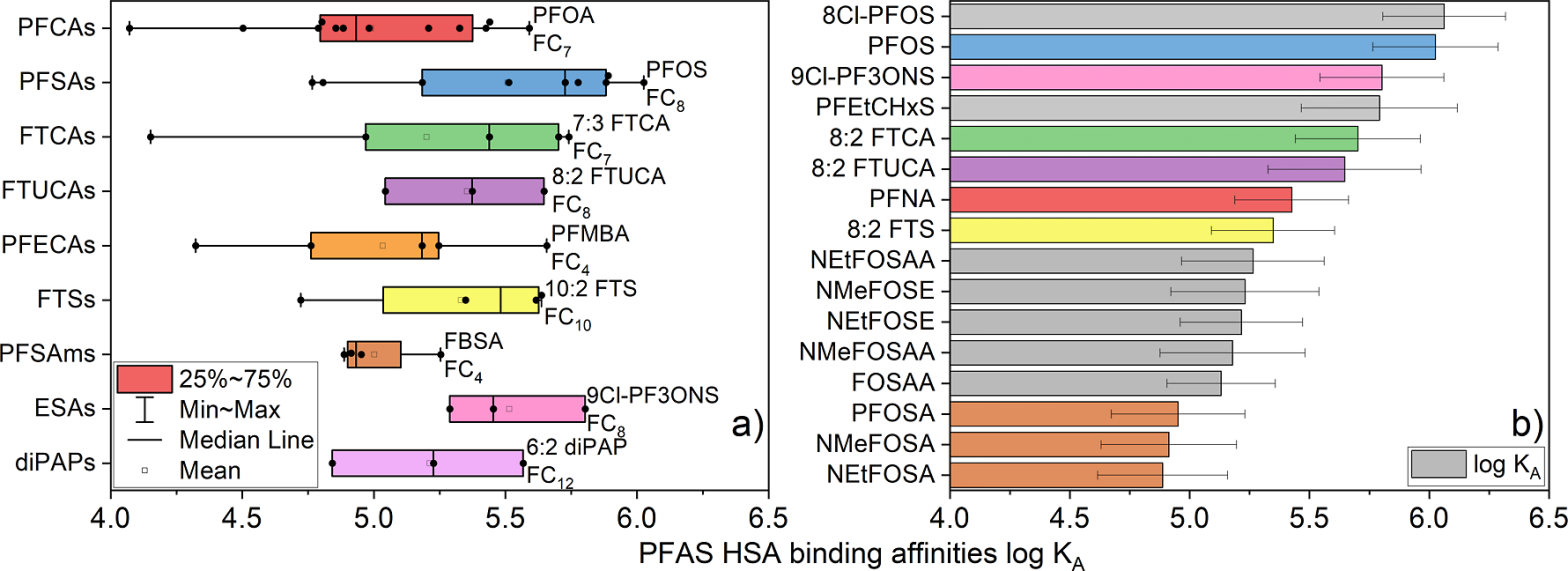
HSA binding affinity for PFAS. (a) Grouped log *K*_A_ based on the structures of PFAS. Each small dot represents the log *K*_A_ for a PFAS in the group. The names and FC_*n*_ of PFAS with the highest binding affinities in each group are denoted to the right of the box. (b) Measurements of perfluorinated PFAS with 8 fluorinated carbons (FC_8_). The FC_8_ PFAS not included in the groups on the left are denoted in gray. PFEtCHxS, compared to PFOS, has two fewer fluorine atoms and likely has lower surface area.

The in vitro HSA binding results showed binding affinities following the order: PFSAs > FTSs ≈ ESAs > FTCAs > FTUCAs > diPAPs ≈ PFECAs > PFCAs > PFSAms, based on log *K*_A_ median values (Figure 2a). The sulfonic acid groups are stronger acids than carboxylic and phosphonic acid groups, indicating that differences in binding for a given chain length are more impacted by the headgroup. These results are consistent with recent bioaccumulation research correlating dust and human serum PFAS concentrations that demonstrated perfluorohexanesulfonic acid and PFOS accumulated in tissues at a higher level than other PFSAs and PFCAs from FC_3_ to FC_12_.^43^ However, given the variations in environmental concentrations of PFAS, tracing the distribution patterns of different PFAS structures from external environments to tissue needs further confirmation. To observe the effect of the FC_*n*_ chain length on binding affinities, the highest values in each group of PFAS are highlighted. Chain lengths of FC_7_ and FC_8_ PFAS were high within the group, and the highest binding among all structure groups followed the sequence: PFOS > 9Cl-PF3ONS > 7:3 FTCA > 8:2 FTUCA ≈ 10:2 FTS ≈ PFMBA > PFOA ≈ 6:2 diPAP > FBSA. The HSA binding patterns differ from the phospholipid membrane partitioning trend, where longer perfluorinated PFAS exhibited stronger partitioning. Similar results are observed in tissue distribution and in both laboratory and field bioaccumulation studies, which are influenced by phospholipid membrane, protein fractions, and binding strengths.^7,44^ The strongest binding for diPAPs occurred with the shorter chain 6:2 diPAP, which might be attributed to the size exclusion effect as excessively large PFAS may not fit well into the HSA binding pockets.

The group of *n*:2 FTS and *n*:2 or *n*:3 FTCAs has a lower median log *K*_A_ than PFSAs but higher than PFCAs for the same FC_*n*_. For 9Cl-PF3ONS and 11Cl-PF3OUdS, which belong to the ESAs group, it is evident that they have the same strong HSA binding affinity as PFOS and perfluorodecanesulfonic acid (Figure S2). However, the larger number of fluorinated carbon atoms in diPAPs did not translate to binding stronger than that for PFSAs, possibly due to the two alkyl chains. HSA binding might be better optimized for a single chain, and the additional chain may not necessarily enhance the binding strength.

### HSA Specific Binding with FC_8_ PFAS Measured by Equilibrium Dialysis

The binding subset of PFAS with eight fluorinated carbons (FC_8_) is highlighted to illustrate the impact of different structural features (Figure 2b). The highest *K*_A_ was observed for 8Cl-PFOS, where compared to PFOS, the last F on the CF_3_ carbon chain is replaced with a Cl atom. The higher steric and hydrophobic effects but lower electronegativity^45^ of Cl compared to F atoms may serve an important role, much as in the binding of other drugs to HSA.^46^ The comparatively stronger hydrophobic effect results in a lower free energy (Δ*G* = −*RT*ln *K*_A_) during protein binding, corresponding to a higher affinity.

In the case of 9Cl-PF9ONS, which has a structure like 8Cl-PFOS but with an additional ether bond, the presence of the ether results in slightly lower affinity than either 8Cl-PFOS or PFOS. It can be observed from this FC_8_-based analysis that the strongest binding affinities are for sulfonic acids, where the carbon chains are all connected to fluorine or chlorine atoms. The binding affinities of polyfluorinated sulfonic acids cannot be directly estimated based on the number of perfluorinated PFAS with equivalent FC_*n*_. For example, 8:2 FTS has the same number of FC_*n*_ and a longer carbon chain than PFOS and PFNA, but a lower binding affinity potentially due to the two CH_2_ groups between the fluorinated carbon tail and the sulfonic acid group. The binding affinity of other FC_8_ PFAS were not as strong as other FC_8_ PFAS with carboxylic acid and sulfonic acid groups. Among all FC_8_ compounds included in this study, the binding affinities of PFSOAAs, perfluorooctane sulfonamide ethanols, and PFOSA were consistently lower than those of PFAS with sulfonic and carboxylic acid head groups.

### Molecular Docking Results Compared to Equilibrium Dialysis

**Figure 3:**
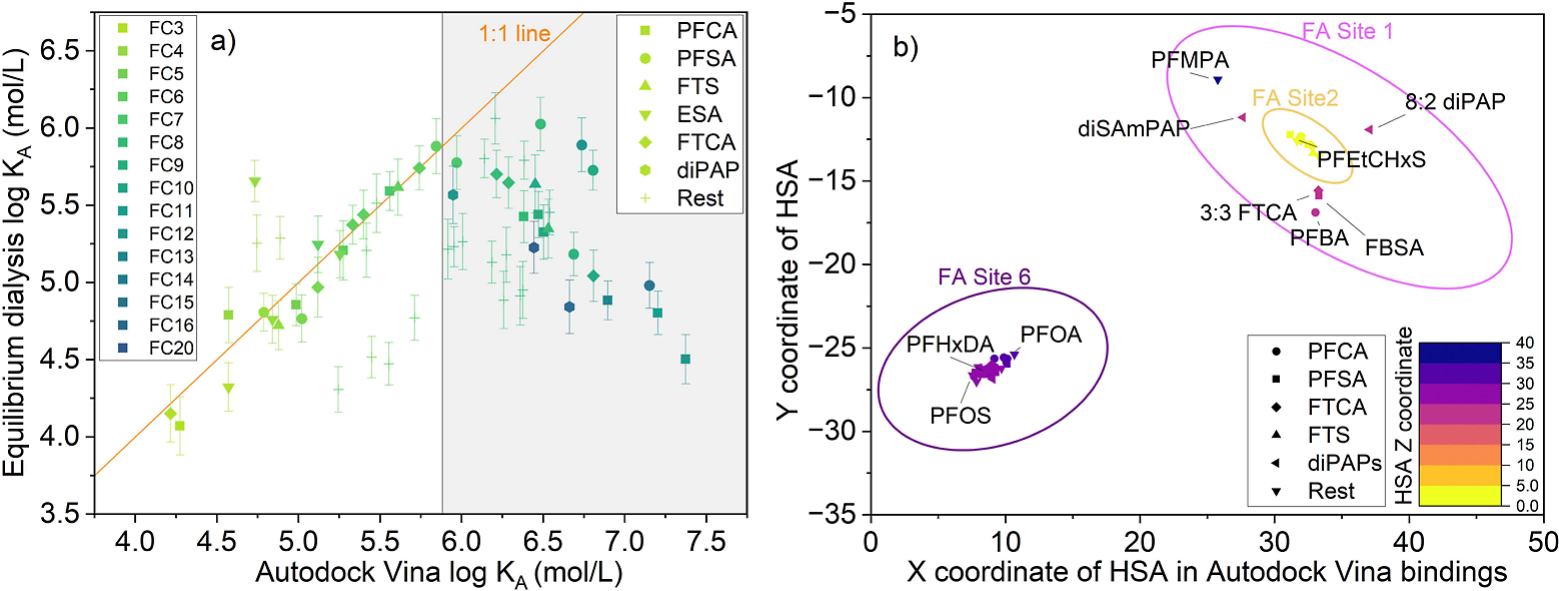
Binding affinity correlations for HSA-PFAS between equilibrium dialysis measurements and molecular docking simulations. (a) The FC_*n*_ count is color-coded from light to dark, while different PFAS groups are represented using different symbols. A noticeable discrepancy emerges between docking results and the measurements within the gray area. (b) Coordinates of PFAS mass centers within the HSA structure for the lowest binding affinity conformation. The unitless *X Y Z* coordinates are from the 1AO6 HSA structure from the PDB database. Locations of FA binding sites (FA 1, 2, and 6) are represented by labeled circles.^25^ To accommodate the graph, the *X*-axis of the docking area is trimmed to remove sections without any PFAS. The *Z* axis of HSA is color-coded, and the colors of box outlines indicate the *Z*-axis center of the docking simulation.

Molecular docking results were compared with equilibrium dialysis measurements to identify the specific active sites on HSA where PFAS are most likely to bind based on their structures. We used AutoDock Vina to predict the HSA–PFAS free energies of binding (Δ*G*, in kcal/mol), and converted predictions to log *K*_A_ using the method reported in previous studies.^22^ The predictions aligned well with equilibrium dialysis for the binding of FC_4_ to FC_8_, when the log *K*_A_ is below 6 (Figure 3a). However, higher modeling results, with log *K*_A_ values from 6.0 to 7.5, are inconsistent with measurements. The discrepancy could result from molecular docking limitations, such as restricted sampling of ligand and receptor conformations, and approximated scoring functions leading to poorer results for longer-chain PFAS.^47,48^ Previous docking results suggest an optimal chain length for significant PFCA-HSA binding and some fundamental interactions, such as the polar carboxylate head interacting with ionizable amino acids (e.g., arginine) while the fluorocarbon tail achieves a minimum energy conformation by associating with hydrophobic residues (e.g., leucine or valine).^49^ We therefore further investigated the predicted PFAS-HSA conformations and binding locations, where the docking predictions for these PFAS were higher than experimental results.

A classification analysis was conducted on the lowest-energy (highest affinity) binding locations of PFAS on HSA (Figure 3b). The highest binding affinities were mostly grouped into three FA sites in HSA’s three-dimensional structure (Figure S3).^25^ PFOA and PFOS were located in FA site 6, which is consistent with the results of the highest binding.^6^ PFAS with FC_3_ to FC_4_ and diPAPs were found in the FA site 1 pocket, and other PFAS were distributed between FA site 1 and FA site 6. This suggests that the predictions from docking may be influenced by the selectivity of the pockets toward the chain length of PFAS and the acidic head groups, leading to the lowest energy conformation not appearing in the same pocket.

Based on these observations, research into binding sites may help explain discrepancies between in vitro equilibrium dialysis experimental results and docking predictions for longer chain PFAS. As surfactants, PFAS may exhibit nonspecific adsorption in equilibrium dialysis experiments (e.g., loss to interfaces). Although the experimental process attempts to minimize this discrepancy through blanks and spikes, it is not eliminated. Regarding the predictions of Autodock Vina, the model places PFAS directly into the active pockets without simulating the process of PFAS having to diffuse through the structure from an external solution as in in vitro experiments. In an in vitro assay, diffusion limitations and the interactions with assay components as PFAS equilibrate with the protein could potentially lead to PFAS being bound at lower energy binding sites first or even participating in multiple site binding, thereby causing the model predictions to be overestimated relative to experimental results. More comparisons obtained through further in vitro and in silico research may help reveal the most physiologically relevant results.

### Comparing Phospholipid Membrane Partitioning and HSA Distribution

**Figure 4:**
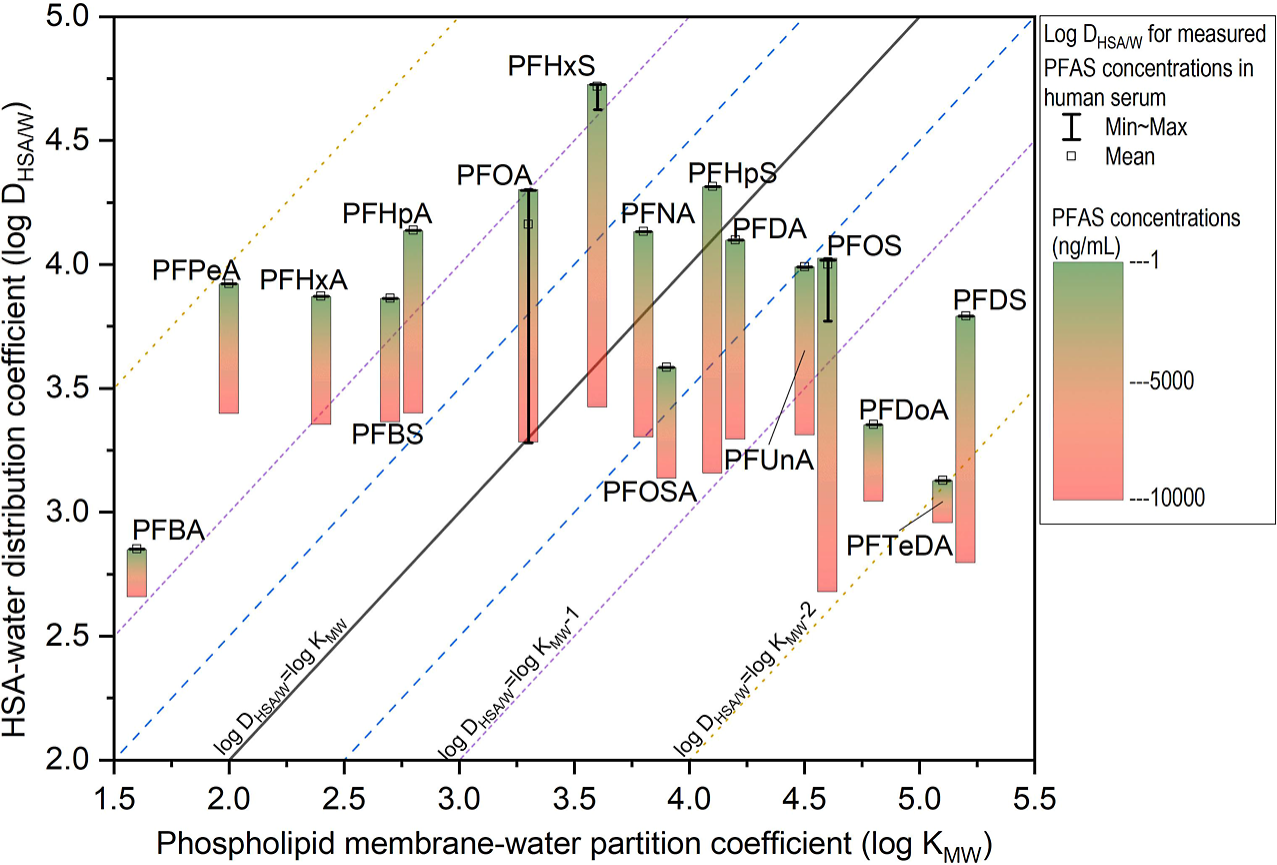
Comparison of PFAS phospholipid membrane–water partition coefficients with HSA/water distribution coefficients based on human serum measurements. The measurement values depicted by the vertical line segments represent the mean PFAS concentrations in human serum, gathered from various international studies.^50-52^ As some PFAS measurements are close, the maximum and minimum values in the distribution might overlap in the graph. The colored box plot illustrates the associated decrease in *D*_HSA/W_ as the concentration of PFAS shifts from 1 to 10,000 ng/mL, transitioning downward in the graph. The placement of PFAS at respective contour levels indicates the relative allocation ratio of PFAS between HSA and the phospholipid membrane at that concentration.

An in silico model was established to study the relative binding strengths for equivalent volumes of phospholipid membrane and HSA, coexisting within a water phase (Figure 4). The PFAS binding affinities between the phospholipid membrane–water and HSA/water were obtained under varying PFAS concentrations within the aqueous phase. Phospholipid membrane–water partition coefficients represent the ratio of the two concentrations; this ratio does not change with PFAS concentrations in the water phase and thus the log *K*_MW_ is a fixed intercept (eq S3). The distribution of PFAS between HSA and water (*D*_HSA/W_) is therefore derived from the PFAS-HSA binding curve (eq 4). The *D*_HSA/W_ for each PFAS decreases when the concentration of PFAS in water increases, which is a feature of saturable specific binding.

The phospholipid membrane partitioning and HSA distribution for different PFAS are compared against a reference of log *D*_HSA/W_ ∼ log *K*_MW_. The modeled concentrations of PFAS in the water phase are based on the levels found in human tissue. The mean concentrations reported for PFAS in human serum ranged from 0.1 to 10,000 ng/mL, when including both occupational and nonoccupational exposure.^50-52^ The PFAS concentrations to the left of the *D*_HSA/W_ = log *K*_MW_ line have log *D*_HSA/W_ values higher than log *K*_MW_. Concentrations between the two dashed blue lines in Figure 4 indicate that the distribution between the HSA and phospholipid corresponds to a one logarithm or factor of 10 difference. Notably for PFAS with a wide measurement range, like PFOA, the *D*_HSA/W_ decreases as concentration increases, leading to different relative values for the two coefficients at corresponding concentration levels—in other words, occupationally exposed populations likely had different internal distributions of PFAS relative to the national background populations. Overall, our analysis indicates that long-chain PFAS tend to bind more to phospholipids, while short-chain ones exhibit a stronger *D*_HSA/W_relative to *K*_*MW*_. No consistent pattern for the difference between *K*_MW_ and *D*_HSA_ across the compounds was found. However, as the concentration rises in the water phase, PFAS exhibits a tendency toward lower *D*_HSA/W_, resulting in a more extensive distribution within the phospholipid membranes. For most PFAS, the reported exposure concentrations are at the low end of this distribution relationship. Thus, the correlation between *K*_MW_ and *D*_HSA_ will be jointly determined by the partition coefficients and the free PFAS in the aqueous phase.

## Supporting Information Available

Definitions of acronyms for PFAS names and groups, additional details on analysis methods, data to support laboratory quality assurance and control, and figures and tables showing all measurement results (PDF)
