# Supplementary material for: Deriving Membrane–Water and Protein–Water Partition Coefficients from In Vitro Experiments for Per- and Polyfluoroalkyl Substances (PFAS)
Source: Environ Sci Technol. 2025 Jan 6;59(1):82–91. doi: 10.1021/acs.est.4c06734 (PMC11740993; doi:10.1021/acs.est.4c06734)
Supplement: Supplementary file 1 — es4c06734_si_001.pdf [file es4c06734_si_001.pdf]

Supporting Information

Deriving membrane-water and protein-water partition coefficients from in vitro experiments for per- and polyfluoroalkyl substances (PFAS)

Ruiwen Chen[1], Derek Muensterman[2], Jennifer Field[2], and Carla Ng[1]*

[1]Department of Civil & Environmental Engineering, University of Pittsburgh, Pittsburgh, PA, USA;

[2]Department of Chemistry, Oregon State University, Corvallis, OR, USA;

[3]Department of Environmental and Molecular Toxicology, Oregon State University, Corvallis, OR, USA.

*E-mail contact: carla.ng@pitt.edu

Pages 27
Figures 8
Tables 5

Experimental design for membrane-water partitioning

The phospholipid membrane-water partition coefficients (K_{MW}) for PFAS used in this study was measured using the TRANSIL Membrane Affinity Kit (Sovicell GmBH, Leipzig, Germany). The solid supported lipid membrane (SSLM) assay immobilizes individual membrane bilayers onto porous silica beads, enabling one side of the phospholipid membrane to face the water phase. The test kits were received frozen and immediately stored at -20 °C until use. Each plate of the kits used in this work contains 500 μL vials distributed in 96-position racks arranged in an 8 × 12 grid, that were comprised of 12 groups, with each group consisting of 8 test tubes. The 8 tubes in each group include 2 blanks and 6 tubes with phospholipid membranes bound to beads at varying volumes of phospholipid membranes (0.067, 0.133, 0.266, 0.529, 1.058, and 2.166 μL), all filled with 500 μL of phosphate-buffered saline (PBS) buffer solution.

Membrane-water partition coefficients (K_{MW}) were measured according to a recently developed experimental procedure.[1] One or more rows of test tubes were thawed at room temperature for 20 min in a ventilation hood before the experiment. Because the PBS buffer solution and phospholipid membrane beads settled at the bottom of the test tube, the tubes were gently shaken to suspend the beads. The suspended liquid was then transferred to 1.5 mL polypropylene centrifuge tubes, and the original vials were rinsed with 500 μL 10mM ammonium acetate, combining the two buffers. After gently shaking, low-speed centrifugation was performed at 500 ×g for 5 min to precipitate the phospholipid membrane beads. Then, 800 μL of the supernatant was discarded and replaced with 800 μL of 10mM ammonium acetate and centrifuged at 500 ×g for 5 mins. Centrifuging and replacing with the new buffer was repeated twice; the new buffer system contains approximately 0.8 % of the initial PBS content. Names, acronyms, Chemical Abstracts Service (CAS) number of the PFAS and group names used in the experiment are listed in Table S1. For membrane-water partitioning experiments, 60 PFAS were divided into groups (Table S1) by structures to reduce the extent of experimentation and minimize the consumption of experimental materials. We evaluated the experimental results for single compounds (PFOA, PFOS, and 6:2 FTS) and their mixtures.

The results showed that the differences were within a log K_{MW} of 0.1, the measurements launched in groups reduce consumption of the experimental resources.

The concentration for each PFAS in a group was prepared at different levels. One of the most studied compounds PFOA was chosen as the typical for other PFAS, all concentrations were conducted in accordance with the ratios of PFOA concentration. The concentration ratio (PFAS μg/L : PFOA μg/L) is listed in Table S2. The PFOA standard was prepared at two concentrations: 100 μg/L for the high level and 10 μg/L for the low level. Each SSLM test series includes 8 tubes: 6 contain volumes of phospholipid, 1 is reserved for a blank, and 1 for a spike control. For the high concentration series, 100 μL of PFOA at a concentration of 100 μg/L was added to the sample tube and spike, while 100 μL of 10mM ammonium acetate was added to the blank. For the low concentration series, 100 μL of PFOA at a concentration of 10 μg/L was added to the sample tube and spike, with 100 μL of 10mM ammonium acetate added to the blank. These tubes were then placed on a laboratory rocker and balanced for 4 hours. After equilibrating, the experimental centrifuge tubes were subjected to centrifugation at 15000 ×g for 10 min. A 200 μL aliquot of the 1000μL supernatant was collected and analyzed by high-performance liquid chromatography-tandem mass spectrometry (HPLC-MS/MS). Three parallel test groups were set up for the two concentration levels (for concentrations for each PFAS see Table S2).

Membrane-water partition coefficient (K_{MW}) measurements

The membrane-water partition coefficient calculation includes direct measurements for PFAS concentrations in water and the indirect estimations in the phospholipid membrane at pH6 and pH7 (Figure S1-S2, Table S5). A spike sample without lipid was set to represent the total amount of PFAS. The mass weight (as μg) of PFAS in the phospholipid membrane was estimated by subtracting the total amount in the aqueous phase from the total mass of the experimental system (eq S1, unit change required to keep K_{MW} unitless).

$$K_{MW} = \frac{C_{PFAS,M}}{C_{PFAS,W}} = \frac{(C_{spike} - C_{PFAS,W}) \cdot V_W}{C_{PFAS,W} \cdot M_{PL}} \qquad \text{eq S1}$$

where,

$C_{PFAS, M}$ is the PFAS concentration in phospholipid membrane (PFAS μg/kg lipid);

$C_{PFAS, W}$ is the PFAS concentration in water phase (PFAS μg/mL);

C_{spike} is the PFAS concentration in water phase of the sample without phospholipid membrane when the experiment ends (PFAS μg/mL);

V_W is the water volume (mL);

and M_{PL} is the mass weight of phospholipid membrane in sample (μg).

However, for the highly hydrophobic groups, due to low solubility in water, they tend to remain on the inner surface of the container, thus the aqueous sample without the phospholipid membrane used in the total mass calculation does not represent the total mass and leads to an underestimation of the PFAS content in the phospholipid membrane. Thus, an additional experimental calculation is required to account for PFAS absorbed from the spike. This extra step involves rinsing the vial with 200 μL methanol, analyzing the PFAS mass in that rinsate, and then combining it with the PFAS measured in the water phase. The difference in PFAS originally added relative to what was measured at the end of the experiment in both the supernatant and rinsate indicates the mass within the phospholipids. Consequently, for PFAS up to and including FC_{10}, the concentration in the phospholipid membrane was calculated in relation to the concentration in the aqueous solution.

$$K_{MW} = \frac{C_{PFAS,M}}{C_{PFAS,W}} = \frac{Mass_{sample\ total} - Mass_{sample\ in\ water} - Mass_{sample\ on\ surface}}{C_{PFAS,W} \cdot M_{PL}}$$

$$= \frac{(Mass_{spike\ water} + Mass_{spike\ on\ surface}) - Mass_{sample\ on\ surface} - Mass_{sample\ in\ water}}{C_{PFAS,W} \cdot M_{PL}}$$

$$= \frac{(C_{spike} - C_{PFAS,W}) \cdot V_W + M_{CP} - M_{CS}}{C_{PFAS,W} \cdot M_{PL}} \qquad \text{eq S2}$$

where,

M_{CS} is PFAS methanol wash down mass weight for the sample from the container surface (PFAS μg);

M_{CP} is PFAS methanol wash down mass weight for the spike from the container surface (PFAS μg);

$C_{PFAS, M}$ is the PFAS concentration in phospholipid membrane (PFAS μg/kg lipid);

$C_{PFAS,W}$ is the PFAS concentration in water phase (PFAS μg/mL);

C_{spike} is the PFAS concentration in water phase of the sample without phospholipid membrane when the experiment ends (PFAS μg/mL);

V_W is the water volume (mL);

and M_{PL} is the mass weight of phospholipid membrane in sample (μg).

Upon preparing all data points for concentrations in the phospholipid membranes and those in water, the partition coefficients are obtained through linear fitting.[1]

$$\text{Log } C_{lipid} = \log C_{aq} + \log K_{MW} \qquad \text{eq S3}$$

Equilibrium dialysis on Rapid Equilibrium Dialysis (RED) system

The RED system was tested as a potentially simpler and higher-throughput method to evaluate HSA-PFAS interactions. The incubation method was based on the research of Gao et al.[2] In brief, the HSA solution was diluted with a buffer solution (0.1 M ammonium acetate, pH=7.4) to 1 μmol/L, and PFAS standard solutions were diluted with the same buffer to a series of 400 μL solutions at 0.0625, 0.125, 0.25, 0.5, 1, and 2 μmol/L. HSA and PFAS standard solutions were prepared on either side of the RED analysis devices. Tests were performed in triplicate. First, 200 μL of HSA was added to the RED HSA tube chamber, and 400 μL of PFAS solution were added to the buffer chamber to achieve equal heights. Second, the equilibrium dialysis device was moved to a shaker at room temperature with a speed of 250 rpm for 4 h to achieve equilibrium. Third, 50 μL of each post-dialysis sample from the buffer chamber was transferred to a 1.5 mL centrifuge tube, 50 μL of methanol was added, the sample was vortexed to mix, and then transferred to a vial for PFAS analysis.

We were unable to obtain specific binding curves for all PFAS with HSA using this system, possibly due to high non-specific adsorption of the more surface-active long-chain PFAS to assay materials, similar in recent work.[3] We therefore focused our equilibrium dialysis work on Slide-A-Lyzer devices (described in the next section), which provide a relatively larger volume and smaller surface area-to-volume ratio compared to RED.

Equilibrium dialysis on Slide-A-Lyzer dialysis devices

Liquid HSA was stored at 4 °C upon receipt. To prepare for the experiment, a 20mM ammonium acetate solution was used to dilute the HSA solution to a concentration of 15 μmol/L (HSA "stock" buffer). For the equilibrium dialysis, the experimental concentration of HSA was prepared with10 mM ammonium acetate buffer solution with specific pH values (pH 7.4) to give a concentration of 1.0 μmol/L. The PFAS water solution prepared in groups as the same as membrane-water partition coefficients measurements section. For PFOA as a typical compound, the stock solution was diluted to 828.1 μg/L (equivalent to 2 μmol/L) in 10 mM ammonium acetate, the highest concentration used in the experiment. A stepwise dilution with buffer was followed to achieve concentrations of 0.0625, 0.125, 0.25, 0.5, 1, and 2 μmol/L. To initiate the experiment, 1000 μL of the PFOA solution was added to the base of polypropylene sample vials. These vials were then fitted with Slide-A-Lyzer vessels with molecular weight cutoff (MWCO) membranes of 10K Da. Next, 100 μL of 1.0 mM HSA was added to each vessel, and the vessels were capped. The whole equilibrium dialysis apparatus was then securely sealed with Parafilm (Bemis, Neenah, WI, USA) and positioned in a shaker. The equilibration process took place at 50 rpm for 48 h. Samples were weighed before and after equilibration to verify that the solution lost was less than 0.001 g to ensure no water evaporation. Finally, 100uL of methanol was added to the sample in the caps, establishing a ratio of aqueous phase to methanol ratio of 1:1 (v:v), and transferred to an LC-MS sample vials for testing (Figure S3, Table S5).

The experimental blank was obtained by placing 1.0 μM HSA in the upper vessel and with ammonium acetate buffer only in the bottom vial. The equilibrium dialysis test series was prepared using a constant 1.0 μM HSA in upper vessels and a series of concentrations of PFAS in the bottom vials. For PFAS controls, the series concentration of PFAS solutions were added to the bottom vials and only blank buffer solution to the upper dialysis vessel. The pH of the buffer solution was adjusted with ammonia or acetic acid. The pH values tested were 6.0 and 7.4. Triplicates for each pH were executed.

For a one site specific binding curve:

$$y = \frac{B_{max}x}{K_D + x}$$

$$y = \frac{N_{binding\ PFAS}}{N_{HSA}}$$

where N refers to the number of moles of bound PFAS and of HSA.

Thus, at a specific PFAS concentration in the aqueous phase (x or C_{aq}), then

$$D_{HSA/W} = \frac{C_{binding\ PFAS}}{C_{aq}} = \frac{N_{binding\ PFAS}}{V_{HSA}} \cdot \frac{1}{C_{aq}} = \frac{N_{binding\ PFAS}}{\frac{N_{HSA} \cdot MW_{HSA}}{\rho_{HSA}}} \cdot \frac{1}{C_{aq}}$$

Thus,

$$D_{HSA/W} = \frac{B_{max} \cdot \rho_{HSA}}{(K_D + \frac{C_{aq}}{MW_{PFAS}}) \cdot MW_{HSA}} \quad \text{(eq S4)}$$

where $D_{HSA/W}$ is the distribution coefficient of PFAS between HSA and water under specific conditions; B_{max} is the total number of binding sites derived from the specific binding curve of a single site; ρ_{HSA} is the density of HSA in g/ml; K_D is the equilibrium dissociation constant in μmol/L; C_{aq} is the concentrations of PFAS in the water phase in g/L; MW_{PFAS} is the molecular weight of PFAS in g/mol; and MW_{HSA} is the molecular weight of HSA in Da/mol.

HPLC-MSMS analytical method

The delay column was a Thermo Scientific (Waltham, MA, USA) Hypersil GOLD C18 , 4.6 × 50 mm, 2.6 μm, and the analytical column was a Thermo Scientific Accucore RP-MS, 2.6 μm, 2.1 × 100 mm, column box temperature: 45 °C, mobile phase A: DI water containing 20 mM ammonium acetate, mobile Phase B: methanol. Sample injection volume: 10 μL. LC gradient was as follows, 0.5 mL/min at 5% B for 0.5 min, then 40% B for 2.0 min at initial, increased B to 95% at 12 min, raised flow speed to 0.45mL/min at 15min, then switched back to initial conditions at 16 min.

The mass spectrometry ion source uses electrospray ionization (ESI). The ion source spray needle temperature was 325 °C, the ion source temperature was 300 °C, and the sheath gas, aux gas, and sweep gas were set to 50, 10, and 1 unit flow speed (Arb), respectively. MSMS transitions are provided in Table S3.

Method validation, quality control and quality assurance

A matrix-matched calibration curve—featuring a water/methanol mixture matrix with standard PFAS concentrations of 0.02, 0.05, 0.2, 0.5, 2, 10, 50, and 100 ng/mL in triplicate—was crafted to assess the method's linearity. The method's lowest quantification (LOQs) estimated from a range of low-concentration standard solutions—spanning from 0.02 to 0.2 ng/mL—spiked into the solvent. The limits of detection (LODs) were the concentrations where the signal-to-noise ratio equaled 3:1.

The calibration curves exhibited linearity ($R^2 > 0.990$). The LOQs spanned a range from 0.2 to 2 ng/mL. Three whole method blank samples were prepared alongside each batch and were concurrently arranged within the sequence, revealing no contamination. Check standards (CCVs) were placed at the initiation and end of each batch sequence, with an additional CCV inserted after every 20 samples. The accuracy from CCVs fluctuated between 80.9% and 121.9 %, and the RSDs lower than 20%

Molecular docking for HSA

To verify the best binding pose of PFAS with HSA at various pH values, blind docking procedures were performed using Autodock Vina[4] on a Linux x86_64 operation system, using Autodock Vina v1.2.6. The crystal structure of HSA (Protein Data Bank ID: 1AO6[5]) was selected as it is an unliganded HSA which may be similar to the purified protein purchased for equilibrium dialysis. The ligand structure of 63 PFAS were screened using the CompTox Chemistry dashboard[6] and then prepared using the python Meeko package[7]. A nine-grid box, each measuring 26 × 26 × 26 Å with a spacing of 1.0 Å, was employed to encompass the entire HSA structure for a blind docking procedure. This ensured that simulations covered the main drug-binding sites of HSA and screened all other potential sites for all the PFAS in this work (Table S4). After that, the Lamarckian genetic algorithm (LGA) was applied to seek the best binding site for PFAS in HSA with the default parameters. For each docking procedure, 20 conformations were output, of which the conformation with the lowest binding energy was

selected and analyzed using the Autodock Vina (Figure S3). The HSA site names including fatty acids (FA) binding sites and drug binding sites[8] are demonstrated in Figure S4.

Linear regression for K_{MW} values with the increase of FC_n.

A linear regression was run for K_{MW} values with the increase of FC_n PFCA and PFSA. The unweighted linear fit was performed in R using the lm() function. The results indicated that as PFCA and FC_n increase, the slope within the current test range is 0.361, with a standard error of 0.014, and an R-square value of 0.982. For PFSA, the slope result within the current test range is 0.374, with a standard error of 0.019, and an R-square value of 0.983.

Phospholipid membrane partitioning and HSA distribution

An *in silico* model was established to study the relative binding strengths for equivalent volumes of phospholipid membrane and HSA (Figure S5). Comparisons of each PFAS HSA/water distribution coefficient and phospholipid membrane/water partition coefficients for concentrations of PFAS in water from 0.1 to 10000 μg/L.

Table S1. Perfluoroalkyl substances (PFASs) names, acronyms, Chemical Abstracts Service (CAS) number, group names used in the experiment. The number of carbons in a single carbon chain (straight or branched) is followed by C with the corresponding number. Non-adjacent carbon chains, such as separated by O or S, are divided into different carbon chains (e.g. C3-C3 for HFPO-DA).

**PFAS Name**	**Acronyms**	**CAS**	**Groups**
Perfluorobutanoic acid	PFBA	375-22-4	Perfluoroalkyl carboxylic acids (PFCA)
Perfluoropentanoic acid	PFPeA	2706-90-3	
Perfluorohexanoic acid	PFHxA	307-24-4	
Perfluoroheptanoic acid	PFHpA	375-85-9	
Perfluorooctanoic acid	PFOA	335-67-1	
Perfluorononanoic acid	PFNA	375-95-1	
Perfluorodecanoic acid	PFDA	335-76-2	
Perfluoroundecanoic acid	PFUnA	2058-94-8	Highly Hydrophobic PFCA
Perfluorododecanoic acid	PFDoA	307-55-1	
Perfluorotridecanoic acid	PFTrDA	72629-94-8	
Perfluorotetradecanoic acid	PFTeDA	376-06-7	
Perfluorohexadecanoic acid	PFHxDA	67905-19-5	
Perfluorobutanesulfonic acid	PFBS	375-73-5	Perfluoroalkyl sulfonic acids (PFSA)
Perfluoropentansulfonic acid	PFPeS	2706-91-4	
Perfluorohexanesulfonic acid	PFHxS	355-46-4	
Perfluoroheptanesulfonic acid	PFHpS	375-92-8	
Perfluorooctanesulfonic acid	PFOS	1763-23-1	
Perfluorononanesulfonic acid	PFNS	68259-12-1	
Perfluorodecanesulfonic acid	PFDS	335-77-3	Highly Hydrophobic PFSA
Perfluorododecanesulfonic acid	PFDoS	79780-39-5	
1H,1H, 2H, 2H-Perfluorohexane sulfonic acid	4:2FTS	757124-72-4	Fluorotelomer sulfonic acids (FTS)
1H,1H, 2H, 2H-Perfluorooctane sulfonic acid	6:2FTS	27619-97-2	
1H,1H, 2H, 2H-Perfluorodecane sulfonic acid	8:2FTS	39108-34-4	
1H,1H,2H,2H-Perfluorododecanesulphonic acid	10:2 FTS	120226-60-0	Highly Hydrophobic FTS
Perfluorooctanesulfonamide	PFOSA	754-91-6	Perfluorooctane sulfonamides (PFOSAm)
N-methyl perfluorooctanesulfonamide	NMeFOSA	31506-32-8	
N-ethyl perfluorooctanesulfonamide	NEtFOSA	4151-50-2	
N-methyl perfluorooctanesulfonamidoacetic acid	NMeFOSAA	2355-31-9	Perfluorooctane sulfonamidoacetic acids (PFSOAA)
N-ethyl perfluorooctanesulfonamidoacetic acid	NEtFOSAA	2991-50-6	
Perfluorooctanesulfonamidoacetic acid	FOSAA	2806-24-8	
N-methyl perfluorooctanesulfonamidoethanol	NMeFOSE	24448-09-7	Perfluorooctane sulfonamide ethanols (PFOSE)
N-ethyl perfluorooctanesulfonamidoethanol	NEtFOSE	1691-99-2	
Hexafluoropropylene oxide dimer acid	HFPO-DA	13252-13-6	Per- and Polyfluoroether carboxylic acids (PFECA)
4,8-Dioxa-3H-perfluorononanoic acid	ADONA	919005-14-4	

PFAS Name	Acronyms	CAS	Groups
Perfluoro-3-methoxypropanoic acid	PFMPA	377-73-1	
Perfluoro-4-methoxybutanoic acid	PFMBA	863090-89-5	
Nonafluoro-3,6-dioxaheptanoic acid	NFDHA	151772-58-6	
Perfluoro(2-ethoxyethane)sulfonic acid	PFEESA	113507-82-7	Ether sulfonic acids (ESA)
9-Chlorohexadecafluoro-3-oxanonane-1-sulfonic acid	9Cl-PF3ONS	756426-58-1	Highly Hydrophobic Ether sulfonic acids (ESA)
11-Chloroeicosafluoro-3-oxaundecane-1-sulfonic acid	11Cl-PF3OUdS	763051-92-9	
3-Perfluoropropyl propanoic acid	3:3FTCA	356-02-5	Fluorotelomer carboxylic acids (FTCA)
2H,2H,3H,3H-Perfluorooctanoic acid	5:3FTCA	914637-49-3	
3-Perfluoroheptyl propanoic acid	7:3FTCA	812-70-4	
2-(Perfluorohexyl)ethanoic acid	6:2 FTCA	53826-12-3	
2-(Perfluorooctyl)ethanoic acid	8:2 FTCA	27854-31-5	
2-(Perfluorodecyl)ethanoic acid	10:2 FTCA	53826-13-4	Highly Hydrophobic FTCA
6:2 fluorotelomer unsaturated carboxylic acid	6:2 FTUCA	70887-88-6	Fluorotelomer unsaturated carboxylic acid (FTUCA)
8:2 Fluorotelomer unsaturated carboxylic acid	8:2 FTUCA	70887-84-2	
6:2 Fluorotelomer phosphate diester	6:2 diPAP	57677-95-9	Phosphate diester (diPAP)
8:2 Fluorotelomer phosphate diester	8:2 diPAP	678-41-1	
EtFOSE-based phosphate diester	diSAmPAP	2965-52-8	
Perfluorohexane sulfonamido amine	PFHxSaAm	50598-28-2	Other PFAS
6:2 Fluorotelomer sulfonamide betaine	6:2 FtSaB	34455-29-3	
N-Trimethylammoniopropyl perfluorohexane sulfonamide	N-TAmP-FHxSA	70248-51-0	
5:3 Fluorotelomer betaine	5:3 FTB	171184-14-8	
5:1:2 Fluorotelomer betaine	5:1:2 FTB	171184-02-4	
8-Chloro-perfluorooctanesulfonic acid	8Cl-PFOS	777011-38-8	
Perfluoro-p-ethylcyclohexylsulfonic acid	PFEtCHxS	646-83-3	
Perfluorobutanesulfonamide	FBSA	30334-69-1	
Perfluorohexanesulfonamide	FHxSA	41997-13-1	

Table S2. PFAS mixture solution composition as ratios relative to PFOA (µg/L : µg/L).

Acronyms	Concentration Ratio (PFAS µg/L:PFOA µg/L)
PFBA	4
PFPeA	2
PFHxA	1
PFHpA	1
PFOA	1
PFNA	1
PFDA	1
PFUnA	5
PFDoA	5
PFTrDA	5
PFTeDA	5
PFHxDA	5
PFBS	1
PFPeS	1
PFHxS	0.914
PFHpS	1
PFOS	0.928
PFNS	1
PFDS	5
PFDoS	5
4:2FTS	4
6:2FTS	4
8:2FTS	4
10:2 FTS	5
PFOSA	5
NMeFOSA	1
NEtFOSA	1
NMeFOSAA	1
NEtFOSAA	1
FOSAA	1
NMeFOSE	10
NEtFOSE	10
HFPO-DA	4
ADONA	3.78
PFMPA	4
PFMBA	3.56
NFDHA	4
9Cl-PF3ONS	3.74
11Cl-PF3OUdS	3.78
PFEESA	4
3:3FTCA	2
5:3FTCA	10
7:3FTCA	10
6:2 FTCA	2
8:2 FTCA	2

**Acronyms**	**Concentration Ratio (PFAS µg/L:PFOA µg/L)**
10:2 FTCA	2
8Cl-PFOS	1
PFEtCHxS	1
FBSA	1
FHxSA	1
6:2 FTUCA	1
8:2 FTUCA	1
6:2 diPAP	5
8:2 diPAP	5
diSAmPAP	5
PFHxSaAm	1
6:2 FtSaB	1
N-TAmP-FHxSA	1
5:3 FTB	1
5:1:2 FTB	1

Table S3. Peak retention times, MSMS transitions, precursor and product ions, collision energy, and LOQ/LOD for HPLC-MS/MS analytical method

Compound	Retention Time (min)	Precursor (m/z)	Product (m/z)	Collision Energy (V)	LOQ (μg/L)	LOD (μg/L)
PFBA	5.51	213.0	169	8.03	0.8	0.229
PFMPA	6.17	229	85	9.6	0.8	0.229
PFPeA	7.22	263	68.9, 219.1	18, 8.8	0.4	0.114
PFBS	7.62	299	80, 99	31.8, 27.3	0.2	0.057
3:3FTCA	7.64	241.1	117.1, 177.1	31.5, 5.3	0.4	0.114
PFMBA	7.7	279.1	85	10.5	0.712	0.203
PFEESA	8.26	315	83, 135	18.4, 20.8	0.8	0.229
NFDHA	8.44	295.1	85.2, 201.1	23.5, 5.3	0.8	0.229
4:2 FTS	8.52	327.1	80.5, 307.1	25.3, 17.6	0.2	0.057
PFHxA	8.62	313.1	119, 269.1	19.8, 7.6	0.2	0.057
PFPeS	8.87	349	80.1, 99	37.3, 28.6	0.2	0.057
HFPO-DA	8.94	285	119, 169	16.5, 5.4	0.8	0.229
FBSA	9	298	78, 119	25, 18	0.2	0.057
PFHpA	9.74	363.1	169.1, 319.1	13.9, 8.5	0.2	0.057
PFHxS	9.85	399	80, 99	33.1, 32.9	0.183	0.052
ADONA	9.86	377	85.1, 251.1	25.7, 9.8	0.756	0.216
5:3 FTCA	9.94	341.1	217, 237	22.1, 10.4	0.2	0.057
5:3 FTB	10.03	414.1(+)[a]	57.9, 104.1	33.1, 27.7	0.2	0.057
5:1:2 FTB	10.2	432.1(+)	58.1, 372.1	35.8, 34.2	0.2	0.057
6:2 UFTCA	10.4	357	242.9, 293.1	33.4, 9.3	0.2	0.057
6:2 FTCA	10.4	377	63.1, 292.9	6, 18	0.2	0.057
6:2 FTS	10.6	427	80.5, 407	28.5, 21	0.2	0.057
PFHpS	10.71	449	80, 99	34.8, 34.6	0.2	0.057
PFOA	11.325	413.1	168.8, 369	15.1, 7.7	0.2	0.057
PFECHxS	11	460.9	99, 381	29, 26	0.2	0.057
6:3 FTCA	11.2	391	267, 287	20, 10	0.2	0.057
FHxSA	11.2	398	78, 378	28, 20	0.2	0.057
N-TAmP-FHxSA	11.3	499.1(+)	59.2, 60.2	40.4, 32.6	0.2	0.057
PFNA	11.36	463	219.1, 418.6	13.6, 9.2	0.2	0.057
PFOS	11.4	499	80.1, 99	39.9, 38.3	0.186	0.053
6:2 FtSaB	11.5	571.1(+)	104.1, 440.1	29, 28.7	0.2	0.057
7:3 FTCA	11.61	441.1	317, 337	19.1, 10.2	0.2	0.057
PFHxSaAm	11.7	485.1(+)	69.9, 85.1	35.7, 31.7	0.2	0.057
9Cl-PF3ONS	11.73	531	350.9, 352.9	23.6, 23.7	0.748	0.214
8Cl-PFOS	11.9	514.9	80, 98.9	41, 40	0.2	0.057
PFDA	11.99	513	218.9, 469.1	16.7, 9.2	0.2	0.057
8:2 FTS	11.99	527.1	80.6, 507	30.5, 23.6	0.2	0.057
8:2 UFTCA	12	457	343, 393	39.3, 11.3	0.2	0.057
8:2 FTCA	12	477	63.1, 393	10, 10	0.2	0.057
PFNS	12.01	549	80.3, 99	43.8, 41	0.2	0.057
FOSAA	12.31	556	419, 497.9	23, 26	0.2	0.057
NMeFOSAA	12.31	570	419.1, 482.9	19.3, 13.7	0.2	0.057
PFUnA	12.48	563.1	269.1, 519.1	15.6, 9.9	0.2	0.057
PFDS	12.48	599	80.1, 98.8	45.9, 42.7	0.2	0.057
NEtFOSAA	12.52	584.1	418.9, 526.1	17.2, 16.9	0.2	0.057
PFOSA	12.63	498	78.1, 478	29.2, 22.4	0.2	0.057
11Cl-PF3OUdS	12.67	630.9	451, 453	26, 25.6	0.756	0.216
PFDoA	12.89	613	319.1, 569	17.3, 9.3	0.2	0.057

Compound	Retention Time (min)	Precursor (m/z)	Product (m/z)	Collision Energy (V)	LOQ (μg/L)	LOD (μg/L)
10:2 FTCA	12.9	577	63.1, 492.9	8, 10	0.2	0.057
10:2 FTS	13.2	627	81, 606.9	34, 29	0.2	0.057
6:2 diPAP	13.34	789	79, 97	38, 28	0.2	0.057
NMeFOSE	14.215	616.1	59.2	13.6	2	0.571
PFDoS	14.085	699	80, 99.4	46.5, 45.4	0.2	0.057
NEtFOSA	13.59	526.1	169, 219.1	24.8, 21.8	0.2	0.057
NEtFOSE	14.3	630	59.2	14	2	0.571
PFHxDA	14	812.9	419, 768.9	20, 12	0.2	0.057
8:2 diPAP	14	989	97.1, 543	30, 20	0.2	0.057
PFTrDA	14.085	663.1	168.9, 619.1	24.9, 10.1	0.2	0.057
PFTeDA	14.21	713	169, 669.1	25.7, 10.3	0.2	0.057
NMeFOSA	14.31	512	169, 218.5	23.7, 22.1	0.2	0.057
diSAmPAP	14.5	1203	168.9, 526	35, 25	0.2	0.057

a. Precursor ions with (+) indicates positive mode for the transition

Table S4: Center positions for protein boxes used in Autodock Vina Docking.

	Center X (Å)	Center Y (Å)	Center Z (Å)
Vina Box 1	28.423	8.39	23.261
Vina Box 2	28.007	-16.292	34.296
Vina Box 3	0.611	-18.589	34.296
Vina Box 4	37.605	-24.137	8.289
Vina Box 5	46.277	-6.186	36.877
Vina Box 6	28.423	34.006	23.261
Vina Box 7	11.643	37.509	43.234
Vina Box 8	51.461	39.786	21.21
Vina Box 9	17.873	25.949	15.926

Table S5: Measurements for membrane water partition coefficients and HSA binding affinities.

PFAS	$\log K_{MW}$@pH7.0	K_D (μmol/L)@pH7.4
PFBA	1.63 ± 0.193	84.9 ± 15.95
PFPeA	2.02 ± 0.149	16.27 ± 3.58
PFHxA	2.42 ± 0.156	13.98 ± 2.79
PFHpA	2.85 ± 0.115	6.2 ± 1.13
PFOA	3.28 ± 0.135	2.57 ± 0.45
PFNA	3.75 ± 0.163	3.75 ± 0.77
PFDA	4.18 ± 0.213	3.64 ± 0.85
PFUnA	4.5 ± 0.215	4.73 ± 1.08
PFDoA	4.82 ± 0.1	15.76 ± 3.34
PFTrDA	5.11 ± 0.187	31.44 ± 5.09
PFTeDA	5.36 ± 0.241	13.09 ± 3.01
PFHxDA	5.73 ± 0.25	10.45 ± 1.71
PFBS	2.72 ± 0.308	15.6 ± 2.68
PFPeS	3.16 ± 0.127	17.15 ± 2.58
PFHxS	3.65 ± 0.115	1.31 ± 0.25
PFHpS	4.09 ± 0.174	1.68 ± 0.36
PFOS	4.5 ± 0.145	0.94 ± 0.23
PFNS	4.82 ± 0.179	6.56 ± 1.36
PFDS	5.15 ± 0.224	1.88 ± 0.4
PFDoS	5.66 ± 0.121	1.29 ± 0.21
4:2 FTS	2.21 ± 0.132	18.96 ± 3.59
6:2 FTS	3.12 ± 0.135	2.42 ± 0.6
8:2 FTS	4.07 ± 0.264	4.49 ± 0.84
10:2 FTS	4.57 ± 0.071	2.31 ± 0.51
PFOSA	3.91 ± 0.162	11.18 ± 1.92
NMeFOSA	4.17 ± 0.172	12.22 ± 1.94
NEtFOSA	4.41 ± 0.23	12.98 ± 2.06
NMeFOSAA	4.21 ± 0.179	6.63 ± 1.49
NEtFOSAA	4.4 ± 0.153	5.44 ± 1.28
FOSAA	4.28 ± 0.257	7.4 ± 1.69
NMeFOSE	3.96 ± 0.12	5.88 ± 0.97
NEtFOSE	4.21 ± 0.089	6.09 ± 1.39
HFPO-DA	2.07 ± 0.129	6.57 ± 1.49
ADONA	2.07	5.68 ± 0.89
PFMPA	2 ± 0.223	47.55 ± 7.91
PFMBA	2.04 ± 0.144	0.22 ± 0.04
NFDHA	2	17.34 ± 3.01
9Cl-PF3ONS	4.38 ± 0.191	1.58 ± 0.39
11Cl-PF3OUdS	4.75 ± 0.263	3.52 ± 0.77
PFEESA	2.49 ± 0.246	5.15 ± 1.22
3:3 FTCA	2.14 ± 0.135	70.57 ± 14.94
5:3 FTCA	2.47 ± 0.1	10.75 ± 2

7:3 FTCA	3.4 ± 0.208	1.82 ± 0.3
6:2 FTCA	2.34 ± 0.103	3.64 ± 0.74
8:2 FTCA	3.23 ± 0.203	1.99 ± 0.37
10:2 FTCA	4.1 ± 0.083	9.06 ± 1.61
8Cl-PFOS	4.73 ± 0.198	0.87 ± 0.17
PFEtCHxS	3.81 ± 0.156	1.62 ± 0.34
FBSA	2.07 ± 0.243	5.58 ± 0.92
FHxSA	3 ± 0.203	3.07 ± 0.57
6:2 FTUCA	2.16 ± 0.023	4.23 ± 0.89
8:2 FTUCA	3.13 ± 0.196	2.26 ± 0.37
6:2 diPAP	4.62 ± 0.18	2.71 ± 0.41
8:2 diPAP	5.17 ± 0.15	14.4 ± 3.11
diSAmPAP	5.06 ± 0.072	5.94 ± 1.1
PFHxSaAm	3.52	6.19 ± 1.01
6:2 FtSaB	2.98 ± 0.2	16.97 ± 3.52
N-TAmP-FHxSA	3.14	33.68 ± 6
5:3 FTB	1.63 ± 0.1	30.44 ± 5.78
5:1:2 FTB	2.01 ± 0.1	49.32 ± 10.1

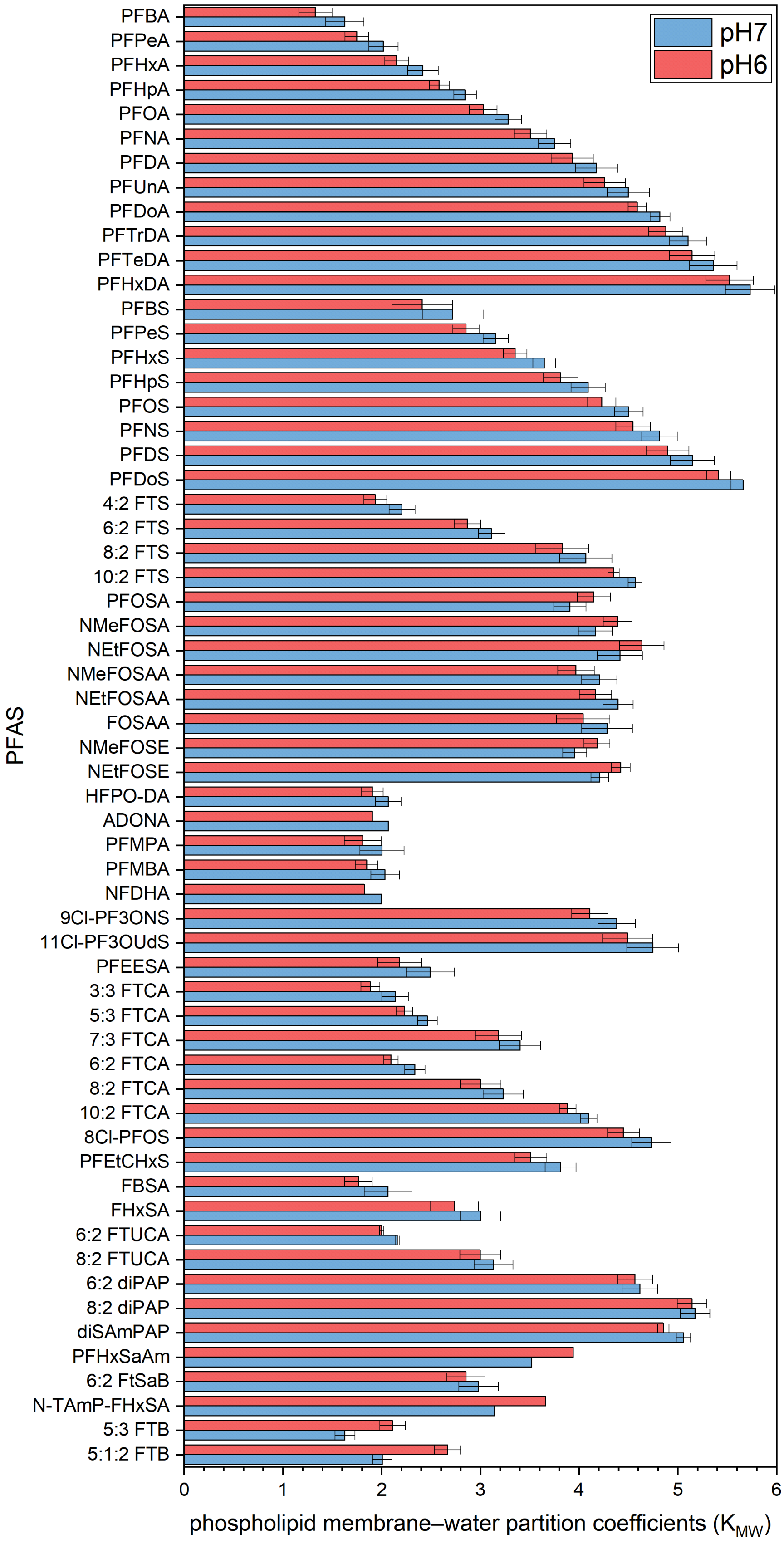

Figure S1. PFAS K_{MW} at pH6 (red) and pH7 (blue).

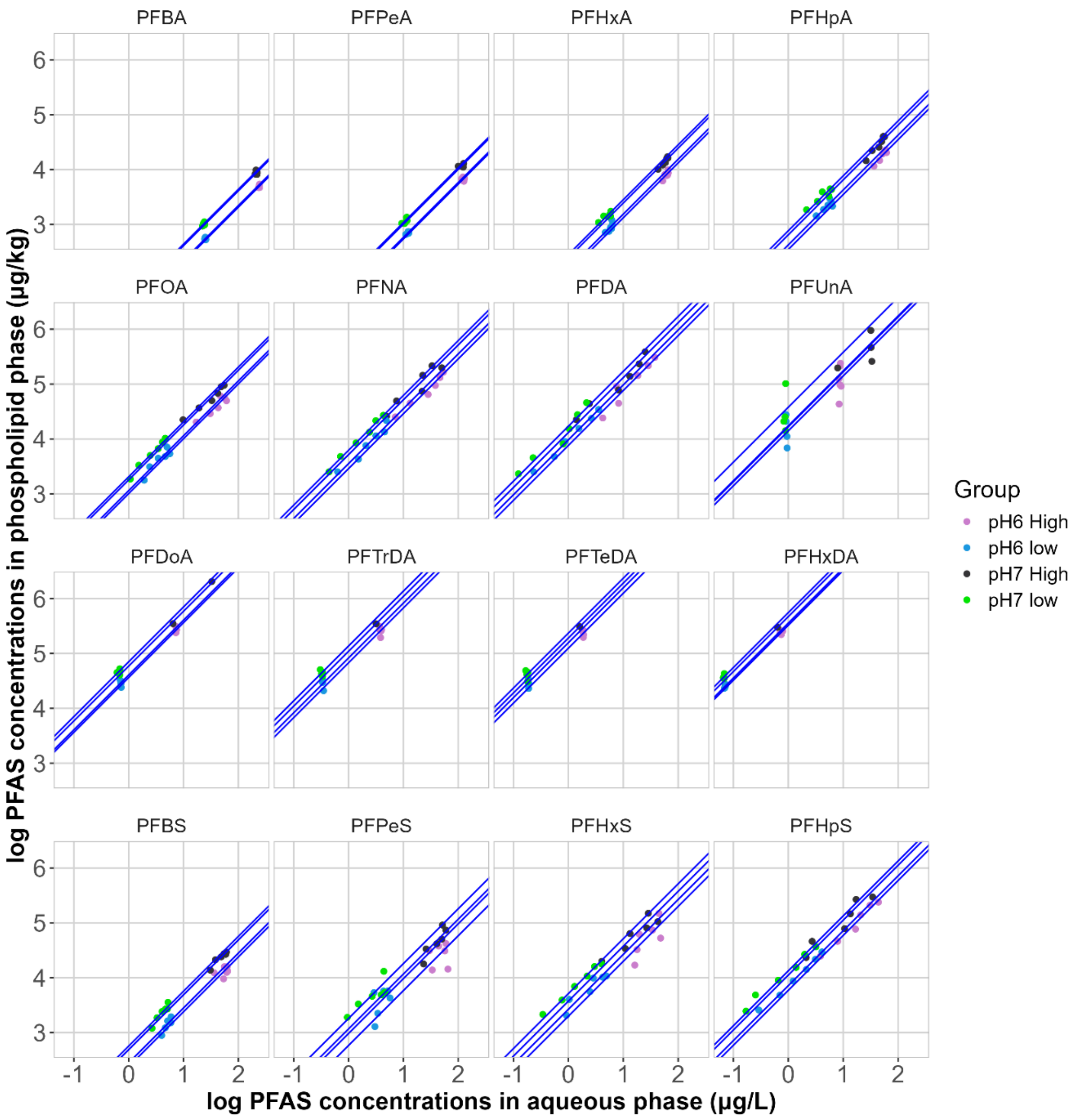

Figure S2. SSLM experimental results for individual PFAS (panel 1 of 4).

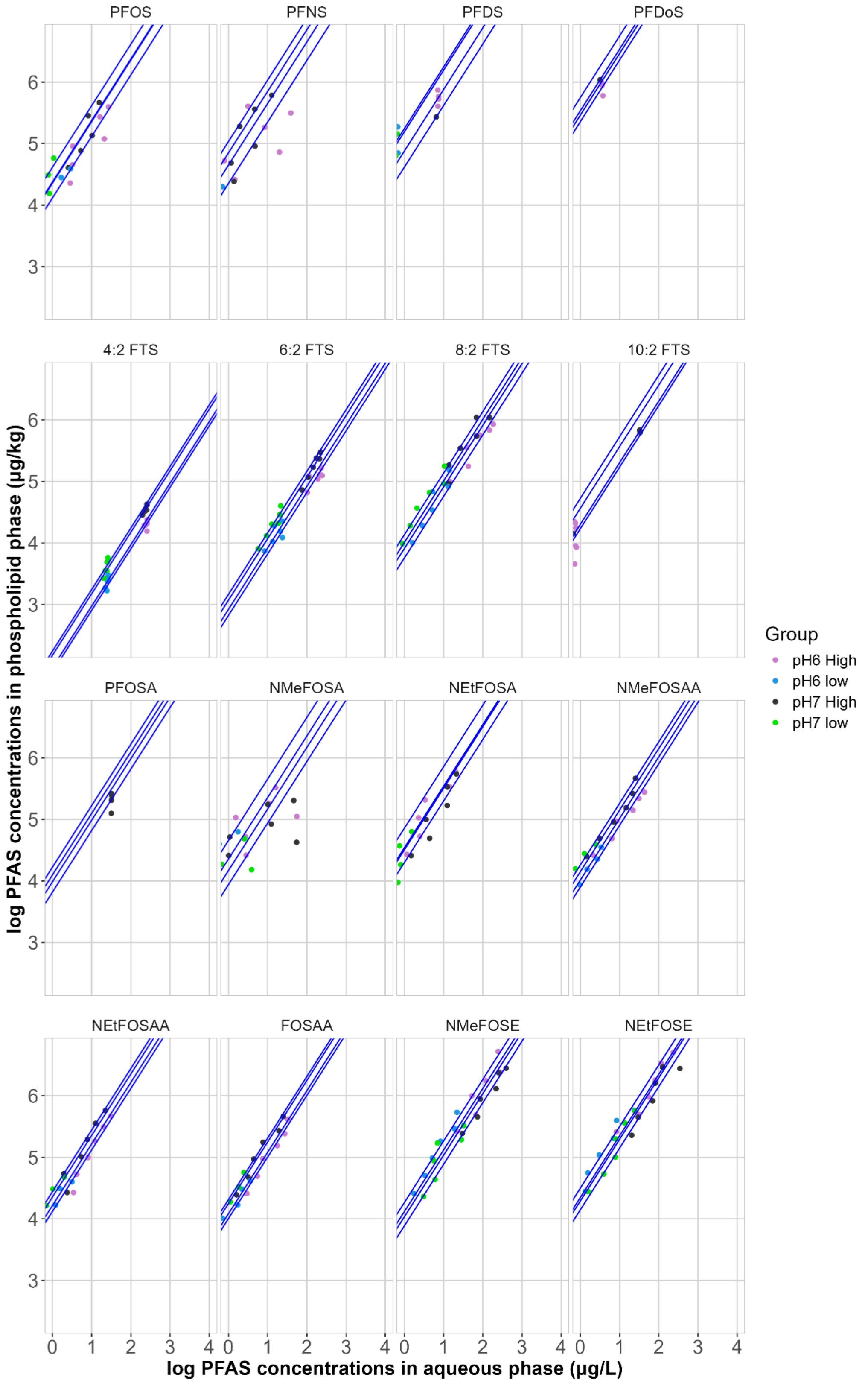

Figure S2. SSLM experimental results for individual PFAS (panel 2 of 4).

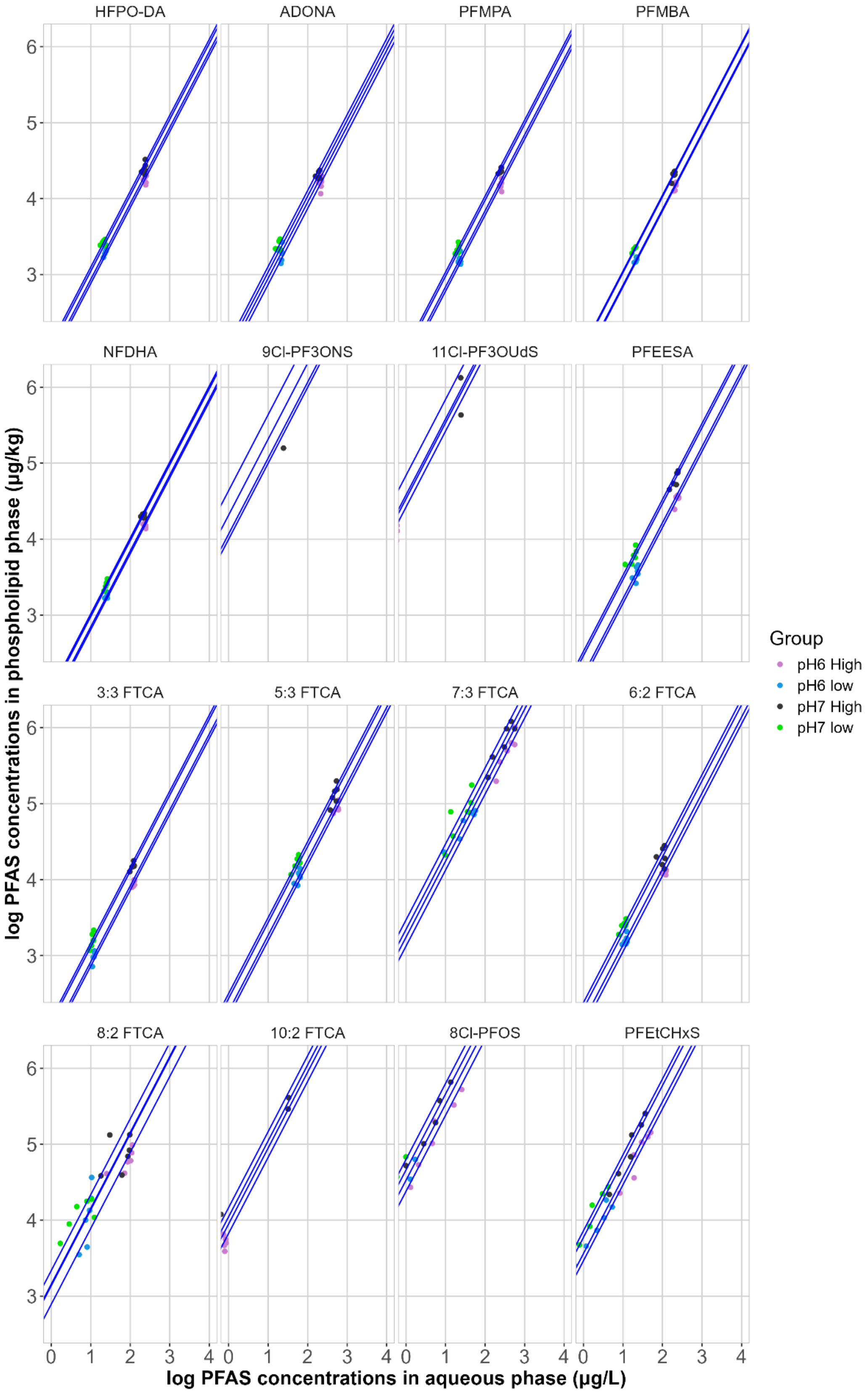

Figure S2. SSLM experimental results for individual PFAS (panel 3 of 4).

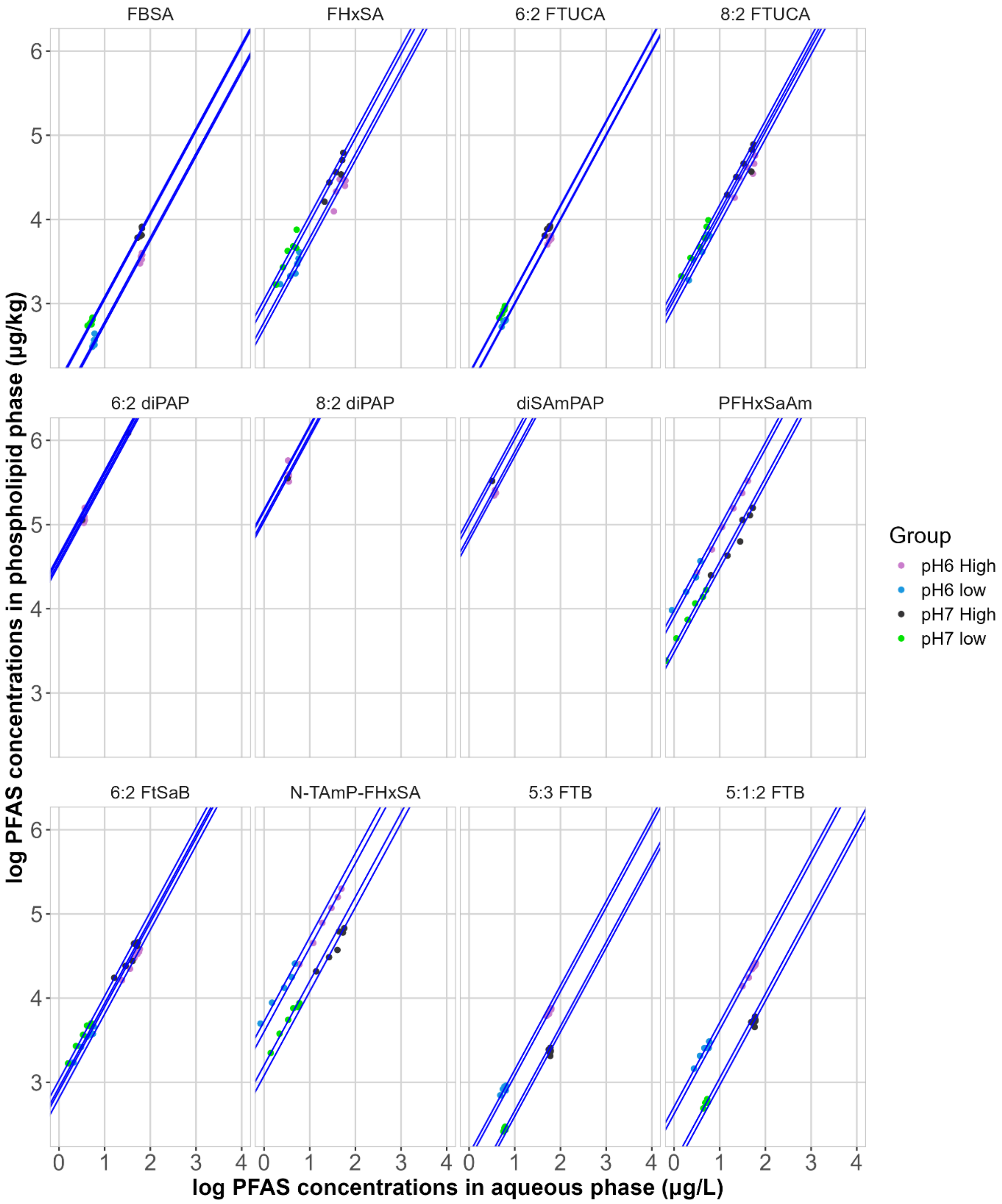

Figure S2. SSLM experimental results for individual PFAS (panel 4 of 4).

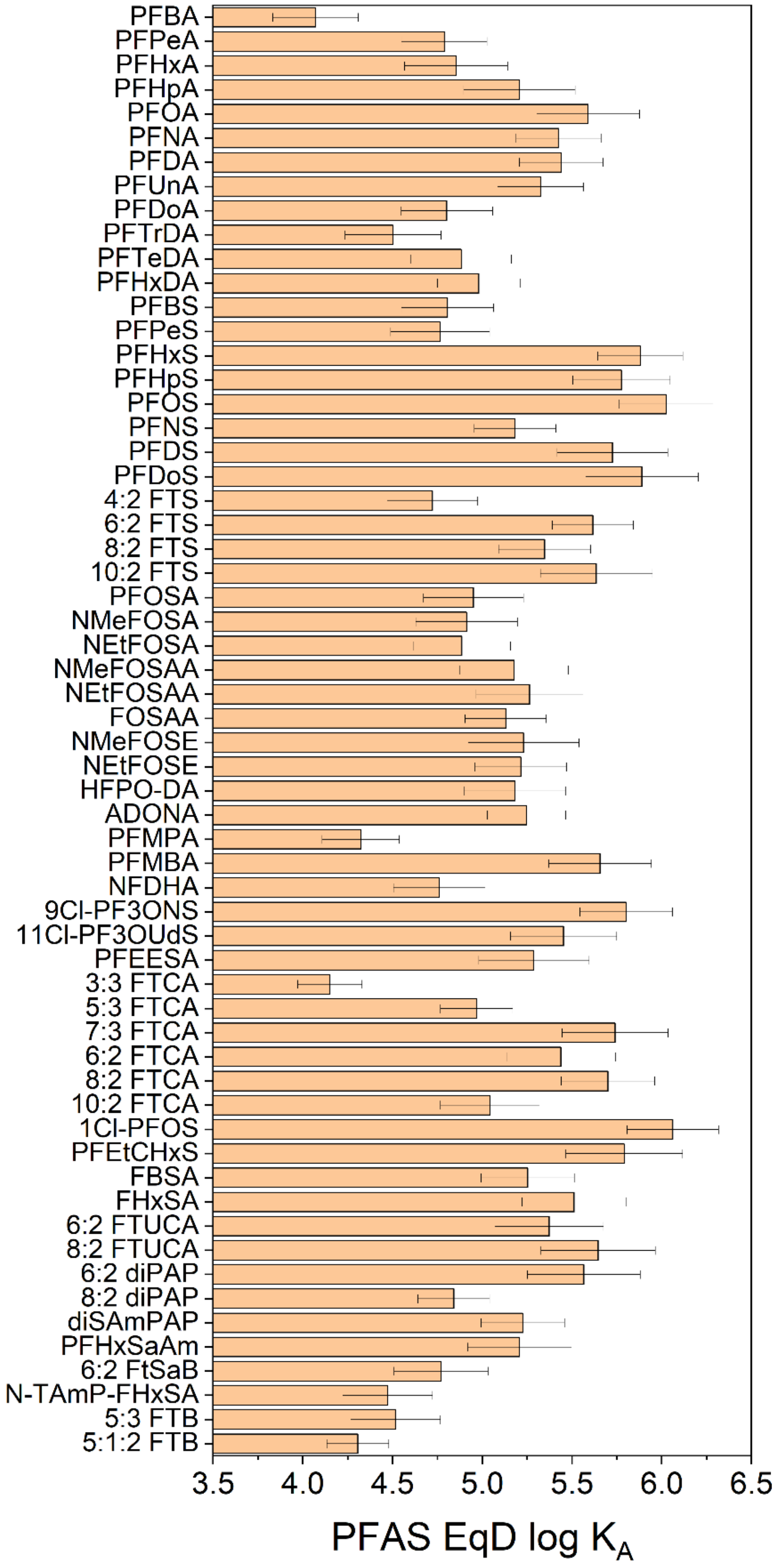

Figure S3. HSA PFAS association constants, K_A.

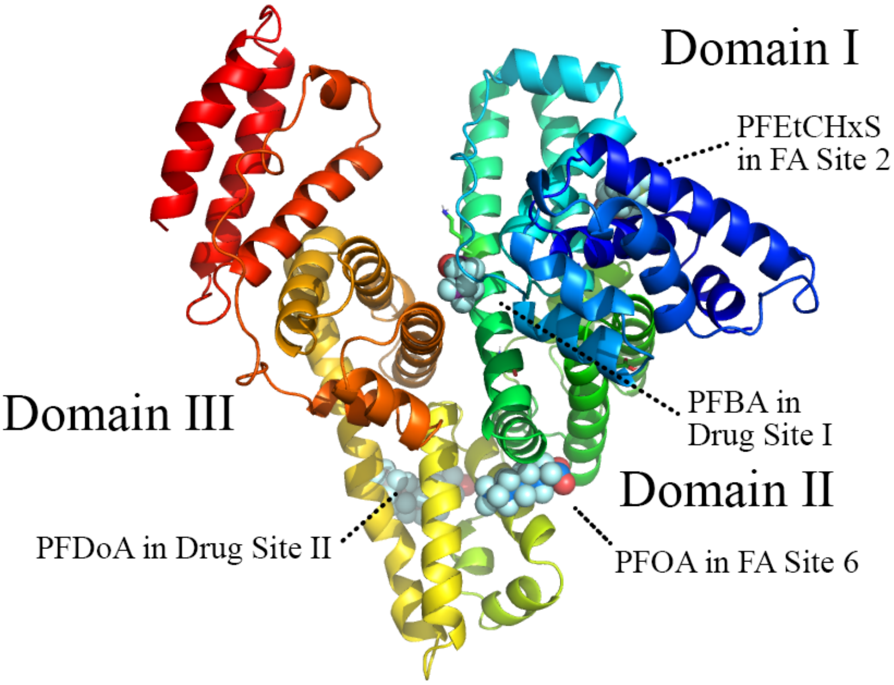

Figure S4. Binding sites for PFAS on HSA (pdb ID: 1AO6). Fatty acid and drug binding sites names were identified and characterized by a recent study.[8]

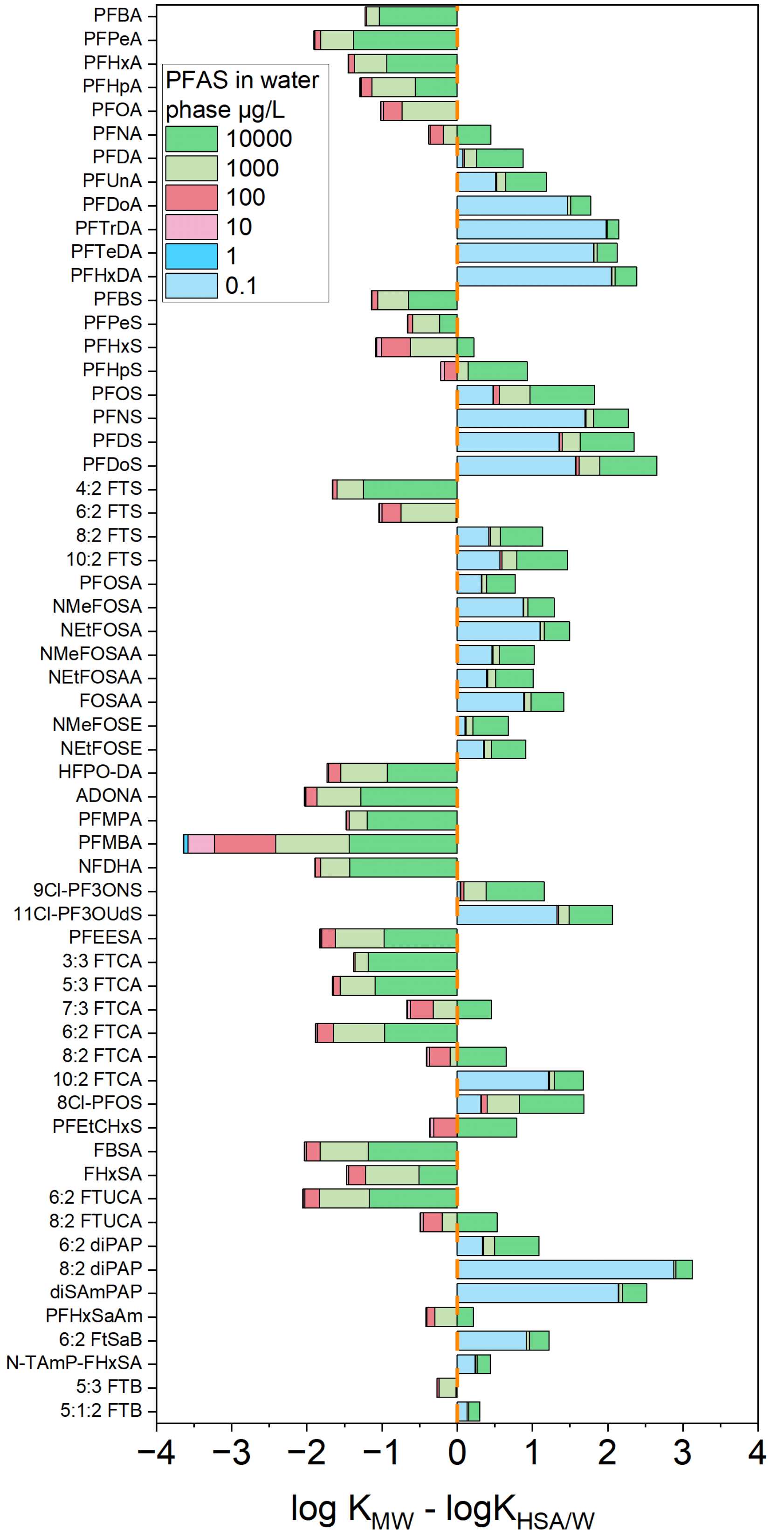

Figure S5. Comparison of HSA/water distribution and membrane/water partition coefficients.

References

(1) Droge, S. T. J. Membrane–Water Partition Coefficients to Aid Risk Assessment of Perfluoroalkyl Anions and Alkyl Sulfates. *Environ. Sci. Technol.* **2019**, *53* (2), 760–770. https://doi.org/10.1021/acs.est.8b05052.

(2) Gao, K.; Zhuang, T.; Liu, X.; Fu, J.; Zhang, J.; Fu, J.; Wang, L.; Zhang, A.; Liang, Y.; Song, M.; Jiang, G. Prenatal Exposure to Per- and Polyfluoroalkyl Substances (PFASs) and Association between the Placental Transfer Efficiencies and Dissociation Constant of Serum Proteins–PFAS Complexes. *Environ. Sci. Technol.* **2019**, *53* (11), 6529–6538. https://doi.org/10.1021/acs.est.9b00715.

(3) Smeltz, M.; Wambaugh, J. F.; Wetmore, B. A. Plasma Protein Binding Evaluations of Per- and Polyfluoroalkyl Substances for Category-Based Toxicokinetic Assessment. *Chem. Res. Toxicol.* **2023**, *36* (6), 870–881. https://doi.org/10.1021/acs.chemrestox.3c00003.

(4) Santos-Martins, D.; Solis-Vasquez, L.; Tillack, A. F.; Sanner, M. F.; Koch, A.; Forli, S. Accelerating AutoDock4 with GPUs and Gradient-Based Local Search. *J. Chem. Theory Comput.* **2021**, *17* (2), 1060–1073. https://doi.org/10.1021/acs.jctc.0c01006.

(5) Sugio, S.; Kashima, A.; Mochizuki, S.; Noda, M.; Kobayashi, K. Crystal Structure of Human Serum Albumin at 2.5 Å Resolution. *Protein Engineering, Design and Selection* **1999**, *12* (6), 439–446. https://doi.org/10.1093/protein/12.6.439.

(6) Williams, A. J.; Grulke, C. M.; Edwards, J.; McEachran, A. D.; Mansouri, K.; Baker, N. C.; Patlewicz, G.; Shah, I.; Wambaugh, J. F.; Judson, R. S.; Richard, A. M. The CompTox Chemistry Dashboard: A Community Data Resource for Environmental Chemistry. *Journal of Cheminformatics* **2017**, *9* (1), 61. https://doi.org/10.1186/s13321-017-0247-6.

(7) Meeko: Preparation of Small Molecules for AutoDock, 2023. https://github.com/forlilab/Meeko (accessed 2023-08-30).

(8) Krenzel, E. S.; Chen, Z.; Hamilton, J. A. Correspondence of Fatty Acid and Drug Binding Sites on Human Serum Albumin: A Two-Dimensional Nuclear Magnetic Resonance Study. *Biochemistry* **2013**, *52* (9), 1559–1567. https://doi.org/10.1021/bi301458b.
